# The State-of-the-Art of Phase II/III Clinical Trials for Targeted Pancreatic Cancer Therapies

**DOI:** 10.3390/jcm10040566

**Published:** 2021-02-03

**Authors:** Andres Garcia-Sampedro, Gabriella Gaggia, Alexander Ney, Ismahan Mahamed, Pilar Acedo

**Affiliations:** Institute for Liver and Digestive Health, Royal Free Hospital Campus, University College London, London NW3 2QG, UK; andres.sampedro.17@ucl.ac.uk (A.G.-S.); gabriella.gaggia.19@ucl.ac.uk (G.G.); alexander.ney.15@ucl.ac.uk (A.N.); ismahan.mahamed.17@ucl.ac.uk (I.M.)

**Keywords:** pancreatic cancer, PDAC, clinical trials, targeted therapies, immunotherapy, cancer vaccines, tumour microenvironment

## Abstract

Pancreatic cancer is a devastating disease with very poor prognosis. Currently, surgery followed by adjuvant chemotherapy represents the only curative option which, unfortunately, is only available for a small group of patients. The majority of pancreatic cancer cases are diagnosed at advanced or metastatic stage when surgical resection is not possible and treatment options are limited. Thus, novel and more effective therapeutic strategies are urgently needed. Molecular profiling together with targeted therapies against key hallmarks of pancreatic cancer appear as a promising approach that could overcome the limitations of conventional chemo- and radio-therapy. In this review, we focus on the latest personalised and multimodal targeted therapies currently undergoing phase II or III clinical trials. We discuss the most promising findings of agents targeting surface receptors, angiogenesis, DNA damage and cell cycle arrest, key signalling pathways, immunotherapies, and the tumour microenvironment.

## 1. Introduction

Pancreatic cancer is a lethal disease with very poor outcomes [1]. Pancreatic ductal adenocarcinoma (PDAC) is the most common pancreatic cancer type accounting for more than 90% of the cases, followed by pancreatic neuroendocrine tumours (PNETs) representing <2% [2]. PDAC five-year survival rate is <9% which falls to 3% for patients with stage IV cancer. Unfortunately, the vast majority of patients are diagnosed at a late stage and pancreatic cancer is predicted to become the second cause of cancer-related death by 2030, only surpassed by lung cancer [3]. There are several factors associated with this poor prognosis, such as the absence of specific symptoms leading usually to late diagnosis, the high resistance of pancreatic cancer cells to available therapeutics, the highly desmoplastic and immunosuppressive tumour microenvironment (TME), the low immunogenicity and importantly, the lack of effective targets for treating early stage disease.

Currently, surgery followed by adjuvant chemotherapy remains the only curative therapeutic option for pancreatic cancer patients, although <20% of patients are resectable at the time of diagnosis (ECOG 0/1) [4]. The main adjuvant chemotherapy used for these patients is a combination of four modified cytotoxic agents: 5-fluorouracil (5-FU), leucovorin, irinotecan and oxaliplatin, also known as mFOLFIRINOX. For patients with advance stage (ECOG 1/2) or metastatic disease (ECOG > 2), first line options consist of a combination of nab-paclitaxel (Abraxane^®^, Celgene, Summit, NJ, USA) plus gemcitabine or mFOLFIRINOX for the most fit patients.

There are three main precursor lesions increasing the risk of developing pancreatic cancer [5]. The most common are pancreatic intraepithelial neoplasms (PanINs), which are microscopic lesions arising from the small intralobular pancreatic ducts. The other two possible drivers are mucinous cystic neoplasms (MCNs) and intraductal papillary mucinous neoplasms (IPMNs). Despite their different origin, whole-genome sequencing studies have shown that their transition from precursor lesions to malignant neoplasms is caused by a first generation of point mutations in the KRAS gene, followed by mutations in tumour suppressor genes such as CDKN2A, TP53 or SMAD4 [6]. Recent studies combining genetic and epigenetic sequencing have identified four distinct pancreatic cancer subtypes based on their molecular signatures (squamous, pancreatic progenitor, aberrantly differentiated endocrine exocrine or ADEX, and immunogenic) [7]. Despite their differences in prognosis, these molecular subtypes have not yet been matched with specific molecular targets that could facilitate therapy selection.

In this regard, pancreatic cancer is known by its wide heterogeneous genetic mutational landscape [8], making the development of personalised and targeted treatment strategies particularly challenging. The detailed molecular characterisation of pancreatic cancer using next-generation sequencing approaches in recent years has allowed the identification of potential molecular targets and the development of novel therapeutic strategies for precision medicine that are currently being tested in clinical trials [9]. These targeted drugs have been designed to interfere with genes and proteins differentially expressed in cancer cells compared to healthy tissue or different components of the TME, inhibiting key factors regulating cancer cell growth, survival, and metastasis [10]. These approaches could minimise side effects associated with conventional therapies by increasing their efficacy and selectivity for the tumour and its stroma.

The high aggressiveness and chemoresistance of pancreatic cancer urge the search for novel and more effective treatment approaches. Targeted therapies will need to overcome some key challenges before becoming first line therapy for this disease. However, several combination strategies with well-established chemotherapeutic agents are currently under development or ongoing. In this review, we have summarised the most promising targeted agents currently in phase II and III clinical trials for pancreatic cancer. The results have been classified taking into account pancreatic cancer landmarks such as surface receptors, molecular signalling pathways, mechanisms for DNA damage repair or cell cycle arrest, and TME components such as the immune system, the tumour stroma, or angiogenic factors. Thus, we will discuss the most relevant and emerging studies already in the clinical setting.

## 2. Targeting Surface Receptors

Receptor mediated drug targeting is a well-established technique to improve the efficacy of drug delivery [11]. Different therapies targeting receptors overexpressed in pancreatic tumours are currently been tested in clinical trials, including zenocutuzumab, also known as MCLA-128, and traztuzumab (both HER-2 inhibitors), cabiralizumab (a Colony Stimulating Factor 1 receptor (CSF1R) inhibitor), and sunitinib (a receptor tyrosine kinase inhibitor).

### 2.1. Human Epidermal Growth Factor Receptor (HER)

**Figure 1:**
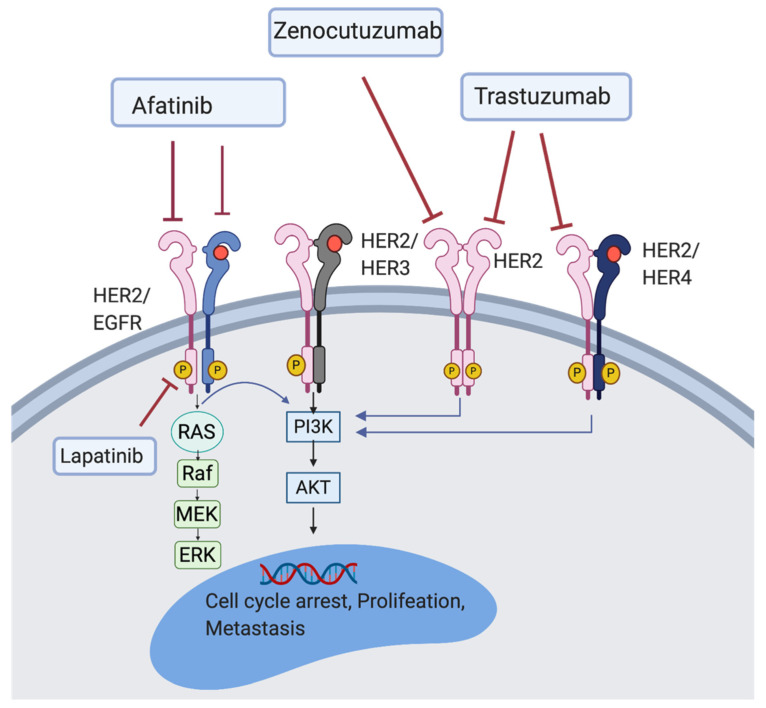
Illustration of the HER2 signalling pathway. HER2 inhibitor (trastuzumab); HER2/EGFR inhibitors (lapatinib and afatinib); HER2/HER3 inhibitor (zenocutuzumab).

HER family plays a critical role in cancer cell proliferation [12]. The HER family is comprised of four main members, HER1-4, present on the cell surface as monomers. When a ligand binds, HER protein dimerise resulting in the autophosphorylation of tyrosine residues which initiate several signalling pathways including mitogen-activated protein kinase (MAPK), phosphoinositide 3-kinase (PI3K), and protein kinase C (PKC) leading to cell proliferation, survival, differentiation, angiogenesis, and invasion [13] (see Figure 1). HER-HER3 heterodimer is the strongest stimulator of downstream pathways, especially the PI3K/AKT signalling pathway which is a main regulator for cell survival and growth [13]. HER2 is also known to be one of the strongest transforming oncogenes.

HER2 targeted therapy has shown significant effect on breast and gastric cancers. In a single arm phase II study including 17 patients with metastatic pancreatic cancer that has failed the first line gemcitabine-based therapy, lapatinib, an inhibitor of both the epidermal growth factor receptor (EGFR) and HER2, was administrated in combination with capecitabin (NCT00881621). However, this study was not completed because of difficulties in enrolment. The median overall survival (OS) was six months [14]. An on-going phase I/II trial using zenocutuzumab (HER2/HER3 inhibitor), has already enrolled 250 pancreatic cancer patients harbouring neuregulin-1 (NRG1) gene fusion (NCT02912949). Moreover, two ongoing clinical trials are investigating the safety and efficacy of traztuzumab, an anti-HER2 monoclonal antibody, in pancreatic cancer (NCT04482309 and NCT04464967). There is also an open label phase I/II study (NCT03602079), using A166, an antibody-drug conjugate targeting HER2 in patients who failed or did not respond to standard pancreatic cancer therapy. A phase I/II trial studying MRTX849 (a KRAS inhibitor) in combination with pembrolizumab, cetuximab (EGFR inhibitor), and afatinib (HER2 and EGFR inhibitor) is also undergoing (NCT03785249). The phase II MATCH trial (NCT02465060), is evaluating the efficacy of genetic testing in patients with different types of solid tumours or lymphomas, including pancreatic cancer. This study aims to enrol 6452 patients altogether over three years, with objective response rate (ORR) as primary and OS and progression free survival (PFS) as secondary outcome measure [15]. The primary aims for all these studies are summarised in Table 1.

### 2.2. Receptor Tyrosine Kinase

Receptor tyrosine kinases (RTKs) regulate several downstream signalling pathways, including MAPK, PI3K/AKT, and JAK/STAT. These pathways play a critical role in cancer stemness, angiogenesis as well as metastasis [16]. In this context, bemcentinib, also known as BGB324 or R428, has been investigated in clinical trials for the treatment of pancreatic cancer. Cabozantinib and sunitinib have also been studied in the clinical setting to target RTK receptors.

#### 2.2.1. Bemcentinib

Bemcentinib (BGB324; R428) is a small molecule targeting the AXL kinase (a member of the Tyro3, Axl, MerTK (TAM) family of RTKs). The AXL receptor tyrosine kinase is overexpressed in approximately 70% of PDAC patients and is associated with metastasis, poor prognosis and chemoresistance [17,18]. By regulating immune cells within the tumour microenvironment, the AXL kinase mediates immune evasion via production of immunosuppressive chemokines [19]. Moreover, AXL expression in tumour cells mediates T cell cytotoxicity resistance, further favouring an immunosuppressive tumour microenvironment. These detrimental effects have sparked explorations into the inhibition of AXL in preclinical and clinical studies. Pre-clinical data have shown that combining bemcentinib with gemcitabine improved gemcitabine efficacy in several xenografts mouse modes of pancreatic cancer [20]. One ongoing phase Ib/II clinical trial is currently assessing the effectiveness of bemcentinib combined with nab-paclitaxel plus gemcitabine and cisplatin for the treatment of patients with metastatic pancreatic cancer (NCT03649321) [21].

#### 2.2.2. Cabozantinib

Cabozantinib is a tyrosine kinase inhibitor that has shown to reduce tumour growth. However, in a phase I trial toxicity was observed when combined with gemcitabine for the treatment of PDAC [22]. The small sample size in this study (*n* = 12) does, however, suggest that further exploration into the safety and efficacy of cabozantinib is required. Despite these disappointing results in PDAC, ongoing phase II and III clinical trials are examining the effectiveness of cabozantinib in PNETs (NCT01466036, NCT03375320) [22].

#### 2.2.3. Sunitinib

Sunitinib is a novel multitargeted RTK inhibitor with antitumour, as well as, antiangiogenic properties. It inhibits at least eight RTK receptors including VEGFR-1-3, CSF1R, and platelet-derived growth factor receptor (PDGFR) a and b [23]. Sunitinib has shown high efficacy and tolerability in the treatment of renal carcinoma and gastrointestinal stromal tumours which led to its FDA approval for the treatment of these two cancers. An international randomised double blinded, placebo-controlled phase III trial testing sunitinib in advanced, well differentiated PNET patients was carried out (NCT00428597) [24]. All patients had Response Evaluation Criteria in Solid Tumours (RECIST) defined disease progression documented within 12 months before baseline. One hundred and seventy-one patients were enrolled in this study, 86 of which received sunitinib and 85 who received placebo treatment. This study was stopped due to side effects and the occurrence of death cases in the placebo group. Authors documented that the median PFS was 11.4 months for patients treated with sunitinib compared to 5.5 months for the placebo group. The ORR was 9.3% in sunitinib treated group compared to 0% in the placebo group.

In November 2010, the European Medicines Agency (EMA) approved the use on sunitinib for the treatment of well differentiated progressed PNET, followed by the approval of the United State Food and Drug Agency (FDA) in 2011. As for PDAC, sunitinib is still in ongoing phase II studies as part of the MATCH trial (NCT02465060). Another randomised phase II trial (NCT02230176) is studying the antitumour effect of ^177^Lu-DOTA^0^-Try^3^-Octreotate (OCLU) versus sunitinib in progressive well-differentiated PNETs.

### 2.3. Colony Stimulating Factor 1 Receptor

The colony stimulating factor 1 receptor (CSF1R) is a cell surface tyrosine kinase receptor expressed by macrophages as well as dendritic cells, neutrophils, and myeloid-derived suppressor cells (MDSCs) [25]. Diverse studies have linked CSF1R with cancer metastasis, invasiveness, and disease progression [26]. CSF1R signalling enhances the recruitment, differentiation, and maintenance of immunosuppressive macrophages into the tumours [27]. PDAC tumours express high levels of colony stimulating factors compared to normal tissues and it has been linked to poor prognosis [28]. In a randomised phase 1a/b trial, patients showed tolerable response to the combination of cabiralizumab (anti-CSF1R) + nivolumab (anti-PD-1). It also showed strong clinical benefit in pre-treated PDAC patients with gemcitabine or 5-FU [27]. In a randomised phase II clinical trial including patients with advanced pancreatic cancer (NCT03336216), patients receive cabiralizumab plus nivolumab or a combination of cabiralizumab, nivolumab, gemcitabine, and nab-paclitaxel. The overall aim is to examine the efficacy of immunotherapy alone versus immunotherapy plus systemic chemotherapy in the treatment of advanced pancreatic cancer.

### 2.4. Erythropoietin-Producing Hepatocellular Receptor 2

The erythropoietin-producing hepatocellular receptor 2 (EphA2) is a member of the mammalian Eph receptor kinase family, which is expressed in epithelial cells and has a role in growth arrest and differentiation. Moreover, by stimulation of cell migration, EphA2 also controls tumour vessel formation [29]. EphA2 overexpression has been observed in pancreatic cancer and associated with poor prognosis. In a non-randomised phase I/II trial (NCT04180371), the BT5528 drug (bicycle peptide targeting EphA2) is being used in combination with nivolumab for the treatment of advanced solid tumours including pancreatic cancer.

### 2.5. Somatostatin Receptor

The somatostatin receptor (SSTR) is expressed in human gastrointestinal tumours, including pancreatic cancer [30]. It prevents angiogenesis and has anti-proliferative effects on both cancerous and healthy cells. There are several clinical trials evaluating SSTR targeting for pancreatic cancer therapy, including a randomised phase III trial (NCT02705651) with 180 patients presenting multiple endocrine neoplasia type 1 (MEN1) PNETs. The aim of this study is to analyse the efficacy of somatostatin analogues on tumour progression. Other clinical trials investigating the use of SSTR in pancreatic cancer are shown in Table 2.

### 2.6. Transforming Growth Factor Receptor

The transforming growth factor beta (TGF-β) is a cytokine of the transforming growth factor family composed of TGF-α and TGF-β. TGF-β signalling deregulation is involved in tumorigenic processes and in the pathophysiology of pancreatic cancer [31,32]. At early stages of pancreatic cancer development, TGF-β acts as a tumour suppressor but at later stages of the disease, it promotes genomic instability, immune evasion and metastasis. A phase Ib dose-escalation and cohort expansion study of safety and activity of the TGF-β inhibitor galunisertib plus the PD-L1 antibody durvaluab, in metastatic pancreatic cancer, reported no dose limiting toxicity and a median PFS of 1.9 months (NCT02734160) [33]. A multi-centre, open label phase Ib/II study (NCT03666832) is investigating the safety, tolerability and efficacy of TEW-7197 (TGF-β receptor kinase ALK5 inhibitor) when combined with FOLFOX chemotherapy, in patients with metastatic pancreatic cancer after failure on gemcitabine and nab-paclitaxel therapy. Some additional receptors currently being targeted for pancreatic cancer therapy are shown in Table 3.

## 3. Targeting Angiogenesis

Initiation of angiogenesis mainly occurs by the interaction of soluble factors with corresponding RTKs located on the endothelial cell surface, ultimately promoting activation of crucial downstream signalling pathways, such as PI3K, PKC, and MAPK [34]. Pro-angiogenic ligands include the vascular endothelial growth factor (VEGF), the fibroblast growth factor (FGF), the platelet-derived growth factor (PDGF) and the epidermal growth factor (EGF). The balance between these pro-angiogenic factors and endogenous angiogenic inhibitors (e.g., angiostatin, endostatin, and arrestin) determines whether endothelial cells remain quiescent or angiogenesis is initiated. Imbalances skewed towards pro-angiogenic factor dominance, as observed in pancreatic cancer, promote an “angiogenic switch” favouring extensive pathological angiogenesis. Despite the hypovascular characteristics of PDAC, production of local pro-angiogenic factors, such as VEGF by cancer cells and stromal pancreatic stellate cells (PSCs), promotes tumour growth and disease progression. PDAC has been associated with overexpression of VEGF, FGF, PDGF, EGF, and their corresponding receptors [34,35]. In particular, overexpression and secretion of VEGF-A by infiltrating M2 macrophages have been shown to facilitate angiogenesis at the tumour periphery following binding to its corresponding receptors—VEGFR-1 and VEGFR-2. VEGF-A is therefore thought to be the main pro-angiogenic mediator in PDAC blood vessel growth.

### 3.1. Targeted Anti-Angiogenic Therapies

**Figure 2:**
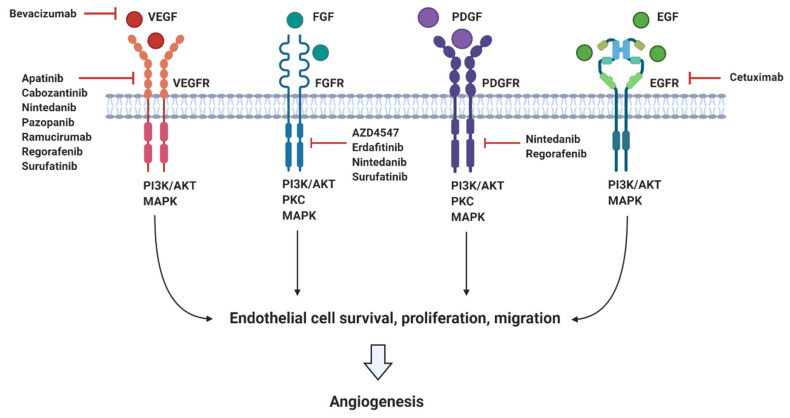
Summary of the main pro-angiogenic factors (VEGF, FGF, PDGF, and EGF), their corresponding receptors (VEGFR, FGFR, PDGFR, and EGFR), and targeted angiogenic inhibitors currently in clinical trials for pancreatic cancer.

There are a vast number of endogenous anti-angiogenic factors including vasohibin and endostatin which directly prevent endothelial cell migration and proliferation [34,35]. Similarly, indirect angiogenic inhibitors have been developed to target pathological angiogenic pathways, preventing the expression or blocking the activity of pro-angiogenic factors. This includes monoclonal antibodies such as bevacizumab, which neutralises VEGF following its secretion from tumour cells, and small molecule inhibitors such as pazopanib, which inhibits the intracellular tyrosine kinase domain of VEGFR to prevent downstream signal transduction following VEGF binding. Other small molecule VEGFR inhibitors include apatinib, nintedanib, regorafenib, and surufatinib. Similarly, small molecule inhibitors of the FGFR (AZD4547, erdafitinib, nintedanib, and surufatinib) and the PDGFR (nintedanib and regorafenib) also exist. In addition, the extracellular domain of angiogenic receptors may be targeted via use of monoclonal antibodies such as ramucirumab, which binds VEGFR to prevent VEGF binding, and cetuximab, which binds EGFR to competitively inhibit EGF binding. A summary of these targeted anti-angiogenic therapies can be seen in Figure 2.

#### 3.1.1. Targeting VEGF

VEGF-A, VEGF-B, VEGF-C, and VEGF-D are all members of the VEGF family. VEGF-A is thought to be the main physiological and pathological pro-angiogenic factor, binding to VEGFR-1 and VEGFR-2 and regulating angiogenesis, vascular permeability, and macrophage and endothelial cell migration [36]. Although VEGF-A binds to VEGFR-1 with a higher affinity than VEGFR-2, intracellular tyrosine kinase activity of VEGFR-1 is 10-fold lower than VEGFR-2 following VEGF-A binding, thus resulting in a weaker downstream response. Specific binding of VEGF-B to VEGFR-1 therefore means that this factor has less of an effect on angiogenesis than VEGF-A, although VEGFR-1 has been implicated in tumour progression [36]. In addition, VEGFR-3 is expressed on lymphatic endothelial cells and is activated by VEGF-C and VEGF-D, playing a large role in the regulation of lymphangiogenesis and tumour metastasis to lymph nodes [36]. VEGF-C and VEGF-D can also activate VEGFR-2 with low affinity and thus, partially stimulate angiogenesis. Overall, VEGF-A and VEGFR-2 are the largest mediators of angiogenesis via downstream activation of the MAPK/PKC/PI3K pathway.

Bevacizumab (Avastin^®^, Roche, Basel, Switzerland) is a monoclonal antibody that targets VEGF-A. It has already received clinical approval for the treatment of several carcinomas including colorectal (2004), lung (2006) and renal (2009) and is undergoing phase I/II clinical trials for the treatment of pancreatic cancer in combination with numerous targeted therapeutics and chemotherapies (see Table 4) [37,38]. One phase II study is currently treating patients with locally advanced/metastatic, unresectable PNETs with everolimus (an mTOR inhibitor) or everolimus plus bevacizumab (NCT01229943). Despite this, previous studies including one phase III trial combining bevacizumab with gemcitabine for the treatment of 535 advanced pancreatic cancer patients showed no clinical significance in improving OS (NCT00088894) [39]. Similarly, another phase III study treating 607 metastatic pancreatic cancer patients with either gemcitabine plus erlotinib (an EGFR inhibitor) and placebo, or gemcitabine plus erlotinib and bevacizumab, showed no statistical significance in improving median OS, although PFS was found to be significantly greater in the bevacizumab arm (NCT01214720) [40]. This may be due to, as observed in other anti-VEGF therapies, the development of innate or acquired resistance resulting from compensatory FGF and PDGF ligand upregulation, thereby elevating downstream MAPK/PI3K signalling and angiogenesis [34,38]. In fact, Casanovas et al. demonstrated FGF upregulation in response to anti-VEGF therapy using a pancreatic cancer mouse model [41]. Sensitivity was restored following simultaneous inhibition of FGF and VEGF [41].

In addition, physiological elevations in the hypoxia-inducible factor-1 α (HIF-1 α) have been shown to allow adaptation of pancreatic cancer cells to hypoxic conditions, further mediating resistance to anti-VEGF therapies [42]. Targeting of HIF-1 α may therefore prove promising in circumventing this resistance and could be beneficial if used alongside bevacizumab. TS-1 (tegafur/gimeracil/oteracil) suppressed HIF-1 α expression following radiation therapy in non-small-cell lung carcinoma tumour xenografts and is currently being explored in phase II trials for the treatment of radiation-treated PDAC patients in combination with gemcitabine (NCT02754180) [43]. Other molecules targeting HIF-1 α have also been explored for the treatment of pancreatic cancer, including TX-2098 [44]. Further clinical studies are however required to determine whether TX-2098 is a viable treatment option for pancreatic cancer.

#### 3.1.2. Targeting VEGFR

VEGFR targeting can be achieved by neutralising antibodies or specific small molecule RTK inhibitors (TKIs) preventing the downstream response elicited by pro-angiogenic factors. Ramucirumab is a monoclonal antibody targeting VEGFR-2 that has recently been approved for the treatment of gastric cancer, and as second-line treatment for non-small-cell lung carcinoma [45,46,47]. However, one meta-analysis determined greater occurrence of serious adverse events following ramucirumab treatment, compared to the control group, across 10 randomised clinical trials [48]. Nevertheless, its efficacy and safety for advanced pancreatic cancer treatment in combination with mFOLFIRINOX is currently being explored in one phase II clinical trial (NCT02581215).

Similar adverse events have been observed with other VEGFR-2 inhibitors. The small molecule inhibitor apatinib, has been associated with increased risk of bleeding in advanced pancreatic cancer when used in combination with TS-1 [49]. Interestingly, one case-study treating a patient with locally advanced pancreatic cancer with apatinib following failed chemotherapy showed promising results with all adverse side effects controlled. Therefore, apatinib is currently undergoing phase II clinical trials for the treatment of metastatic/advanced pancreatic cancer, in combination with the anti-PD-1 immune checkpoint inhibitor camrelizumab (NCT04415385), or with TS-1 and irinotecan (NCT04101929) [50].

The overexpression of multiple angiogenic receptors observed in pancreatic cancer suggests that simultaneous multi-receptor inhibition may be more beneficial than single receptor blockade. In fact, this approach is currently being explored. For example, regorafenib inhibits a group of RTKs including VEGFR, PDGFR, and FGFR. Proven to reduce tumour growth, vascularity, and metastasis, it has been approved as second-line treatment for metastatic colorectal cancer. However, a phase II clinical trial exploring the use of regorafenib for the treatment of refractory metastatic pancreatic cancer has not shown positive results [51]. Further investigations are ongoing with another two-phase II studies evaluating its effectiveness in patients with metastatic solid tumours (NCT02307500) and metastatic NETs (NCT02259725) (see Table 4). Similarly, the VEGFR/FGFR inhibitor surufatinib (HMPL-012; sulfatinib) has been used to treat patients with PNETs. One single-arm phase Ib/II clinical trial, NCT02267967, included 81 PNET patients and showed encouraging results following surufatinib treatment [52], supporting two subsequent phase III clinical trials (NCT02589821 and NCT02588170). Both phase III trials validated the promising results observed in the phase Ib/II study [53].

The triple angiokinase inhibitor nintedanib (targets VEGFR1-3, FGFR1-3, and PDGFR), is being explored in a phase I/II clinical study to treat patients with advanced pancreatic cancer (NCT02902484). Toxicity-related issues were not described in previous trials for prostate and ovarian cancer [54]. Similarly, the efficacy of the oral multi-kinase inhibitor pazopanib (VEGFR1-3, PDGF), to treat pancreatic cancer has been evaluated. One phase II clinical trial utilised pazopanib to treat metastatic gastroenteropancreatic neuroendocrine tumours (NCT01099540) [55]. Results demonstrated a disease control rate of 75.7% and an ORR of 18.9%, with 9 partial responses confirmed. Another phase II study explored the combination of pazopanib with depot octreotide for the treatment of advanced NETs (NCT00454363) [56]. The trial consisted of 52 patients (32 PNETs; 20 carcinoid tumours) with OR observed in 21.9% of PNETs, although none were seen in the carcinoid tumour cohort. Pazopanib is currently being investigated in a phase II clinical trial for the treatment of patients with progressive carcinoid tumours (NCT01841736), and in combination with temozolomide (a DNA alkylating/methylation agent) for the treatment of advanced, unresectable PNETs (NCT01465659).

#### 3.1.3. Targeting FGFR

The FGFR family of receptors include FGFR-1-4. They are RTKs expressed on endothelial cells (except for FGFR-4) and mediate cell proliferation, differentiation, migration, survival, and angiogenesis [57]. There are 18 members forming part of the FGF family—12 of which activate FGFRs. Overexpression of FGFR-2 in particular has been observed in pancreatic cancer, promoting tumour angiogenesis and migration following activation by ligands FGF7 and FGF10 [58]. Although no therapeutics specifically targeting FGF are currently being explored for pancreatic cancer, anti-FGFR therapies such as erdafitinib (an FGFR1-4 inhibitor) are in clinical trials. Erdafitinib is approved for treating locally advanced/metastatic urothelial cancer [59], and is currently being investigated in the phase II MATCH trial (NCT02465060). The effectiveness of the FGFR1-3 inhibitor AZD4547 against pancreatic cancer is also being investigated in this trial.

#### 3.1.4. Targeting EGFR

EGFR overexpression is common in pancreatic cancer patients and is associated with poor prognosis [60]. Cetuximab, an anti-EGFR monoclonal antibody, has been approved for the treatment of colorectal cancer, as well as squamous cell carcinoma of the head and neck, and is in trials for the treatment of advanced pancreatic cancer [61]. One phase II study by Xiong et al. using cetuximab plus gemcitabine to treat EGFR-expressing advanced pancreatic cancer showed promising results with 63% of the 41 patients enrolled achieving stable disease, although subsequent studies have failed to replicate this [62,63,64,65]. A phase III trial treating 745 patients with advanced/metastatic pancreatic cancer (90% EGFR positive) with gemcitabine alone or gemcitabine plus cetuximab, demonstrated no significant differences in response, median survival or PFS. These results have led some experts to propose that cetuximab should no longer be considered as a treatment option for pancreatic cancer [66], although it is still being explored, alongside additional targeted therapies, in two ongoing phase I/II clinical trials. Combinations being explored include cetuximab plus MRTX849, pembrolizumab, and afatinib in the treatment of KRAS G12C-mutant pancreatic cancer (NCT03785249), as well as cetuximab plus trastuzumab and SNK01 for the treatment of advanced, EGFR-positive pancreatic cancer (NCT04464967).

### 3.2. Other Angiogenic Targets

In addition to the above-mentioned pro-angiogenic factors, other targets also contribute to the development of pathological angiogenesis in PDAC. For example, the connective tissue growth factor (CTGF) mediates tissue remodelling and fibrosis but can also promote endothelial cell migration and proliferation. Therefore, one anti-CTGF antibody, pamrevlumab, has been investigated in phase I/II clinical trials for the treatment of locally advanced, unresectable pancreatic cancer in combination with gemcitabine and nab-paclitaxel [67]. Results showed 71% of the pamrevlumab-treated group to be eligible for surgical exploration following treatment (*n* = 24), compared to 15% of patients not receiving pamrevlumab (*n* = 13) [68]. A phase III clinical trial is currently evaluating pamrevlumab plus gemcitabine and nab-paclitaxel for the treatment of locally advanced pancreatic cancer (NCT03941093). Another inhibitor in a phase I/II trial for pancreatic cancer treatment is the endothelin B receptor antagonist, ENB003 (NCT04205227). This receptor is overexpressed in PDAC and promotes endothelial cell migration and proliferation [69].

Although promising, anti-angiogenic therapies have not been effective against pancreatic cancer yet. This may be due to the aforementioned compensatory upregulation of other angiogenic factors, although non-angiogenic mechanisms of vascularisation including vasculogenesis, vessel co-option, and vasculogenic mimicry may also serve as potential barriers to anti-angiogenic therapy effectiveness [34,70].

## 4. Targeting DNA Damage Response

DNA synthesis and replication during cell division are essential for cell proliferation. Defects in these processes may occur due to extrinsic and intrinsic factors leading to DNA damage [71]. Cells have constitutive mechanisms to detect DNA damage. For that reason, the induction of DNA damage has been widely exploited as a promising strategy for cancer therapy, using extrinsic or intrinsic factors that impair DNA replication and activate the DNA damage response (DDR), leading to cell death. There are several types of DNA lesions that can activate the DDR. Radiation therapies for cancer treatment induce breaks in the double helix of the DNA, which can be single strand breaks (SSBs) or double strand breaks (DSBs). In SSBs the remaining undamaged strand acts as a template and guide the repair. However, for DSBs the DDR mechanisms activated are more complex often leading to more effective treatments. In normal conditions, DSBs are repaired via homologous recombination (HR) or non-homologous end joining (NHEJ) [72,73].

Different targeting approaches have been directed against PDAC genetic aberrations with great importance for DDR mechanisms such as the BRCA1, PALB2, BRC2, and RAD51 genes [74]. In a normal cell undergoing DSBs, the MRN protein complex (formed by Mre11, Rad50, and Nbs1) detects strand breaks and interacts with the breast cancer type 1 susceptibility protein (BRCA1) which initiates repair by HR. BRCA1 recruits BRCA2 and the partner and localiser of BRCA2 (PALB2) to the damage site. The complex formed by BRCA1, BRCA2, and PALB2 activates the DNA repair protein RAD51 homolog 1 (RAD51) which starts the break repair [75]. Around 24% of PDAC tumours display mutational patterns such as the BRCAness phenotype (loss of BRCA1/2), PALB2, RAD51, and other genes involved in DSB repair. This PDAC hallmark is currently being tested in several phase II and III clinical trials, evaluating the combination of crosslinking or ionising radiation treatments, which cause DNA breaks, with targeted drugs impairing the DDR.

**Figure 3:**
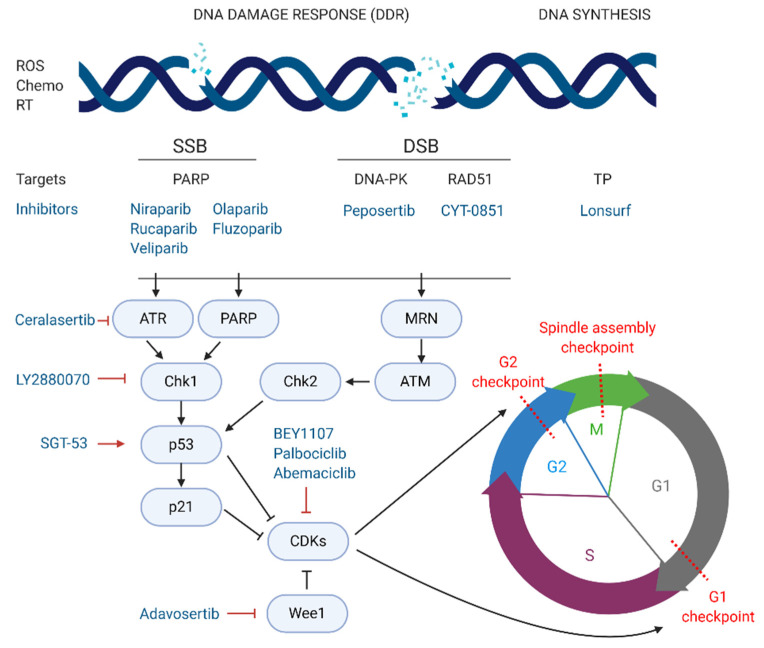
Inhibitors of DNA damage response and cell cycle arrest currently in phase II or III for pancreatic cancer treatment. DDR, DNA, damage response; ROS, reactive oxygen species; Chemo, chemotherapy; RT, radiotherapy; SSB, single strand break; DSB, double strand break.

The most exploited target involved in DNA repair is the family of poly (ADP-ribose) polymerases or PARPs, particularly PARP-1/2 in pancreatic cancer. These nuclear proteins are responsible for the detection of SSB in the DNA and recruitment of the DNA-repair enzymatic machinery. There are several PARP inhibitors currently in phase II and III studies for the treatment of pancreatic cancer (see Figure 3). Their mechanism of action is based on the so called PARP-trapping model, in which PARP is trapped to the DNA helix by the inhibitor, impairing the correct replication of the DNA [76]. Pancreatic tumours harbouring mutations in genes involved in DDR are especially sensitive to PARP inhibition by targeted therapies. Some examples of successful PARP inhibitors are the small molecules niraparib, rucaparib, veliparib, olaparib, and fluzoparib (see Table 5) which differ in their potency to trap PARP to the DNA [77].

Niraparib is been investigated in different phase I/II and II clinical trials for pancreatic cancer treatment. The NIRA-PANC phase II study is testing niraparib in patients with metastatic disease that have previously received first line chemotherapy (NCT03553004) [78]. Its efficacy is being tested specifically for patients bearing germline deleterious or somatic mutations in DDR genes as previously described (NCT03601923). The combination of niraparib with immunotherapeutic agents targeting PD-1, such as dostarlimab (NCT04493060), dostarlimab plus radiotherapy (NCT04409002), nivolumab, or ipilimumab (anti-CTLA-4) (NCT03404960), are currently under investigation, suggesting that the future of pancreatic cancer treatment could take advantage of novel synergistic approaches of immunotherapy and targeted PARP inhibitors.

Rucaparib is another PARP inhibitor in phase II trials for advanced and metastatic pancreatic cancer (NCT03140670, NCT04171700). This agent has been already approved for other cancers such as prostate cancer carrying the BRCA mutation and ovarian cancer. Interestingly, rucaparib is currently being tested in combination with chemotherapy (liposomal irinotecan, fluorouracil and leucovorin) in a phase I/II study, for patients with metastatic pancreatic cancer (NCT03337087).

Veliparib is also being trialled for stage III and IV pancreatic cancer, in combination with standard of care chemotherapy. A phase II multicentre randomised trial compared 50 gBRCA/PALB2^+^ PDAC patients undertaking veliparib alone or in combination with gemcitabine plus cisplatin (NCT01585805). In this case, even though the group receiving the triple combination showed a median PFS slightly higher than the chemotherapy alone (10.1 months), the study did not meet the proposed response rate (RR) endpoint (*p* = 0.55) (17). A different study tested the combination of veliparib with mFOLFIRI compared with FOLFIRI alone in 123 patients with metastatic pancreatic cancer (NCT02890355). However, the results did not show any increase in OS (5.4 vs. 6.5 months) or PFS (2.1 vs. 2.9 months). One last study has reached phase I/II stage testing veliparib with modified 5-fluorouracil and oxaliplatin (mFOLFOX-6) in metastatic pancreatic cancer with BRCA mutations, but no results are available yet (NCT01489865).

Olaparib alone (NCT02677038, NCT02184195) or in combination with pembrolizumab (anti PD-1 agent, NCT04548752), cediranib (VEGF inhibitor, NCT02498613), or ceralasertib (a potent inhibitor of the Ataxia Telangiectasia and Rad3 related (ATR) protein kinase responsible of SSB recognition, NCT03682289), are currently in phase II and III clinical trials. The POLO study (phase III), a multicentre study carried out in 12 different countries, investigated the efficacy of olaparib monotherapy compared to placebo. The study enrolled 154 metastatic patients with genomic mutations in the BRCA genes whose disease did not progress after first line platinum-based chemotherapy. The results showed a median PFS of 7.4 months in the treatment group compared to 3.8 months in the placebo arm (*p* = 0.004), but no significant changes in OS (18.9 months vs. 18.1 months). The olaparib arm also showed some major adverse events including cholangitis (2.20% of patients), abdominal pain (3.30%) and anaemia (6.59%) [79].

Lastly, fluzoparib, a selective PARP1/2 inhibitor, has recently started a phase III study (NCT04300114). The trial is focused on metastatic patients with BRCA1/2 or PALB2 mutations resistant to platinum-based chemotherapy. A separate phase I/II trial is testing the efficacy of fluzoparib plus mFOLFIRINOX compared to mFOLFIRINOX alone for advanced pancreatic cancer (NCT04228601).

An alternative therapeutic strategy to target pancreatic cancer cells consists on inhibiting the correct function of the DNA-dependent protein kinase (DNA-PK), responsible for repairing DSB by NHEJ [73]. Peposertib (also known as nedisertib or M3814) inhibits the ability of DNA-PK to repair DSBs [80]. The combination of peposertib with hypofractionated radiotherapy for locally advanced PDAC, compared to radiotherapy alone, is in phase I/II trials (NCT04172532). As previously mentioned, RAD51 is another key protein for DNA repair of DSBs via HR, and therefore a potential target for pancreatic cancer patients. In the phase I/II multi-centre clinical trial NCT03997968, CYT-0851, a potent inhibitor of RAD51, is currently being tested as a single agent in 14 different malignancies, including locally advanced or recurrent metastatic PDAC. Another strategy for the induction of DNA damage focuses on impairing DNA synthesis. Lonsurf (previously TAS-102), a combination of the drugs trifluridine and tipiracil hydrochloride, has been approved since 2015 for metastatic colon cancer and is now in phase I/II trials, in combination with liposomal irinotecan, for advanced gastrointestinal cancers including stage III and IV pancreatic cancer (NCT03368963).

Overexpression of the serine/threonine kinase, glycogen synthase kinase 3β (GSK-3β), has been associated with reduced survival in PDAC patients, via regulation of the ATR DDR pathway. Moreover, signalling of mutant Kras has been shown to increase GSK-3β expression, which ultimately aids the growth and survival of Kras-mutant tumours [81]. Promising preclinical findings led to the initiation of an ongoing phase I/II clinical trial exploring the small molecule inhibitor 9-ING-41 to inhibit GSK-3β (NCT03678883).

## 5. Targeting Cell Cycle Arrest

The cell cycle is composed of four phases—G1 (growth), S (DNA synthesis), G2 (growth and preparation for mitosis) and M (mitosis), and three strongly conserved cell cycle checkpoints to minimise the accumulation of mutations—(i) at the end of G1 phase, (ii) at the G2/M transition, and (iii) during M phase [82]. Cyclins and cyclin-dependent kinases (CDKs), such as CDK1 and CDK2, constitute the main positive regulators of the cell cycle. Complexes formed by CDKs and cyclins activate, via phosphorylation, proteins controlling the progression of cells through the cell cycle checkpoints. On the other hand, negative regulators such as p53, p21, and the retinoblastoma protein are responsible for the detection of DNA damage and recruitment of DDR repair enzymes. If the damage cannot be repaired, nuclear accumulation of high levels of p53 activates the translation of p21, a major inhibitor of CDK/cyclin complexes, blocking the progression from G1 to S phase and triggering apoptosis [83].

In cancer, including pancreatic cancer, positive cell cycle regulators, such as CDK1, are often overexpressed and linked to poor prognosis [84]. Thus, targeted strategies inhibiting CDKs are being explored as novel treatment options for pancreatic cancer. On the other hand, inhibition of negative regulators of CDKs, such as checkpoint kinase 1 (Chk1) or Wee1 kinase, both responsible for the blockage of CDK/cyclin-dependent cycle progression regardless of DNA damage, also constitute promising targets for PDAC [85,86].

BEY1107, a novel inhibitor of CDK1, is in phase I/II as monotherapy and in combination with gemcitabine for the treatment of locally advanced or metastatic pancreatic cancer (NCT03579836). Another promising targeted agent in clinical trials is adavosertib, an inhibitor of Wee1. Two clinical trials are investigating the potential of adavosertib in pancreatic cancer patients. The NCT02194829 trial is studying adavosertib in combination with nab-paclitaxel and gemcitabine for stage III-IV disease. Similarly, the above-mentioned MATCH trial (phase II) is treating PDAC patients exhibiting the BRCAness phenotype with adavosertib monotherapy (NCT02465060). The same trial is also testing palbociclib, an inhibitor of CDK4/6. In this line, abemaciclib, another CDK4/6 inhibitor is also in phase II for a variety of unresectable and metastatic neuroendocrine tumours, including PNETs, that have not responded to first line therapy (NCT03891784). Lastly, a multicentre phase I/II study carried out in Canada and USA, is testing the inhibition of Wee1-activator Chk1 by the agent LY2880070, in different solid tumours, including advanced and metastatic pancreatic cancer (NCT02632448). The study will assess the efficacy of LY2880070 alone and in combination with gemcitabine. A previous phase Ib study already showed that combination of these two agents allowed reduced dosing due to their synergistic effect [85].

An interesting approach is being tested at the Mary Crowley Cancer Research Center (USA) and the National Taiwan University Hospital (Taiwan) (NCT02340117). In this phase II study, metastatic pancreatic cancer patients receive a combination of nab-paclitaxel, gemcitabine and the gene-therapy agent SGT-53. SGT-53 consists of wild type cDNA of the p53 gene encapsulated in a liposomal formulation, aiming at restoring the wild-type function of p53. The combination of DNA-damaging agents, like gemcitabine or nab-paclitaxel, with the active form of p53 aims at stopping tumour progression via cell cycle arrest and induction of apoptosis.

In addition to the clear function that cyclins, CDKs and their consequent activators and inhibitors, play in cell cycle regulation, there is an upcoming trend to investigate the role of epigenetic modulators in cancer development. Histone deacetylases (HDACs), together with histone acetyltransferases (HATs), are key controllers of epigenetic gene regulation. Overexpression of HDACs has been linked to the development of different cancers including pancreatic cancer. Entinostat, a selective class I HDAC inhibitor, is being tested in combination with nivolumab (anti-PD-1) in a phase II study for patients with metastatic cholangiocarcinoma and pancreatic cancer (NCT03250273). Similarly, romidepsin (or istodax), a natural product obtained from the Gram-negative bacteria *Chromobacterium violaceum*, has also shown anti-HDAC properties and is currently approved for the treatment of other cancers like T-cell lymphomas. Romidepsin, in combination with nab-paclitaxel and gemcitabine, is also in clinical trials for advanced pancreatic cancer (NCT04257448).

Other epigenetic modulators like DNA methyltransferases (DNMTs) represent effective targets for pancreatic cancer treatment [87]. Azacitidine (commercialised as Vidaza and approved for acute myeloid leukaemia treatment) is an analogue of cytidine that has been described to covalently bind DNMT1 to the DNA, blocking its epigenetic function and causing DNA damage. This compound is now in phase II for the treatment of resected pancreatic cancer patients with elevated CA19-9 levels (NCT01845805). Azacitidine is also being tested in another phase II trial in combination with the immunomodulator pembrolizumab (NCT03264404).

## 6. Targeting Signaling Pathways

### 6.1. JAK/STAT Pathway

The Janus-associated kinase-signal transducer and activator of transcription (JAK-STAT) pathway is involved in the development of multiple human cancers [88]. Four JAK and seven STAT family members have been described in humans, although significant elevations in expression and activation of JAK1/2 and STAT3, in particular, have been observed in pancreatic cancer patients [89,90,91]. Overexpression of JAK-STAT pathway components, such as IL-6, EGFR, and Src, have also been seen in pancreatic cancer [87]. Similarly, loss of negative pathway regulators, such as the suppressor of cytokine signalling 1 (SOCS1), further exacerbates JAK-STAT pathway activation [92].

**Figure 4:**
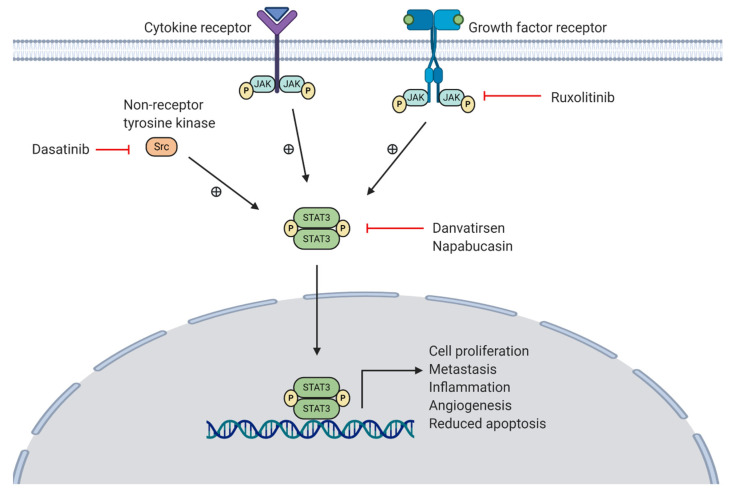
Overview of STAT3 activation via the JAK-STAT pathway (cytokine and growth factor receptor stimulation) and cytoplasmic Src kinase. A JAK inhibitor (ruxolitinib), STAT3 inhibitors (danvatirsen, napabucasin) and a Src inhibitor (dasatinib) are currently in clinical trials for pancreatic cancer.

The activation of the JAK-STAT pathway is mediated via receptor-associated JAK trans-phosphorylation and activation following stimulation of cytokine and growth factor receptors by corresponding ligands, such as IL-6 and EGF (see Figure 4) [93]. This leads to JAK-mediated tyrosine phosphorylation and translocation of STAT to the nucleus [88]. Associated cellular responses include production of IL-6 to mediate inflammation, VEGF to promote angiogenesis, Bcl-xL to inhibit apoptosis, and matrix-metalloproteinases (MMPs) to mediate invasion and metastasis [88]. Although STAT activation is largely facilitated by JAKs, other non-RTKs, such as Src, are also involved [93]. Furthermore, STAT3 has been shown to mediate immunosuppressive effects in the TME via production of IL-10 and TGF-β, supporting tumour growth whilst reducing anti-tumour immunity [93]. Inhibition of these pathway components, particularly STAT3, is therefore considered a promising option for the treatment of pancreatic cancer. An overview of this pathway and the inhibitors currently in phase II (ruxolitinib, danvatirsen, and dasatinib) and phase III (napabucasin) clinical trials can be seen in Figure 4.

#### 6.1.1. JAK Inhibition

Inhibition of JAK to prevent downstream STAT3 activation may reduce cellular proliferation, invasion and metastasis. To date, clinical application of JAK inhibitors, including pacritinib, ruxolitinib, and tofacitinib, has mainly involved chronic inflammatory conditions such as rheumatoid arthritis. However, one phase II clinical trial evaluated ruxolitinib, a selective JAK1/2 inhibitor, for pancreatic cancer therapy (NCT01423604) [93]. This trial included 127 patients with metastatic PDAC and previously failed gemcitabine therapy. Results did not demonstrate significant differences in OS between the two groups (ruxolitinib plus capecitabine or placebo plus capecitabine), although patients with high levels of the inflammatory marker C-reactive protein (CRP) did show significant differences in median survival following treatment with ruxolitinib plus capecitabine (2.7 months versus 1.8 months in those with normal CRP) [94]. Two successive phase III trials (JANUS 1 and JANUS 2) enrolling metastatic pancreatic cancer patients presenting high CRP levels began in 2014, although both were subsequently terminated due to a lack of efficacy seen at interim analysis (NCT02117479 and NCT02119663) [95]. Another phase II study is currently underway (NCT02955940).

#### 6.1.2. STAT3 Inhibition

Hyper-activation of STAT3 has been associated with poor prognosis in pancreatic cancer patients [93]. Therefore, STAT3 inhibition has been extensively explored to reduce tumour growth and metastasis. The therapeutic antisense oligonucleotide danvatirsen (formerly IONIS-STAT3-2.5Rx; AZD9150), inhibits the production of STAT3 by binding to STAT3 mRNA [96]. Preclinical data have suggested that STAT3 inhibition combined with immunotherapy may enhance therapeutic effectiveness and reduce immunotherapy resistance [97]. This was clinically validated in a phase Ib/II clinical trial treating 38 patients with PD-L1-naïve advanced solid tumours and recurrent/metastatic head and neck cancer with danvatirsen combined with durvalumab (an anti-PD-L1 antibody) as second-line treatment (NCT02499328) [98]. These promising results have supported further phase II trials testing this combination therapy in other cancers, including advanced pancreatic cancer (NCT02983578).

Napabucasin (BBI608) is a small molecule inhibitor of STAT3 evaluated for the treatment of several cancers including platinum-resistant ovarian cancer, gastric adenocarcinoma, and pancreatic cancer [99]. A phase Ib/II study tested napabucasin in combination with nab-paclitaxel for pre-treated metastatic PDAC [100]. Results were promising with a response rate of 7%, and a disease control rate of 52% in evaluable patients (*n* = 31). Similarly, another phase Ib/II clinical trial using napabucasin plus nab-paclitaxel and gemcitabine to treat 71 metastatic PDAC patients showed disease control in 92% of the cases and partial response in 43% of the 60 evaluable patients [101]. Moreover, a study by El-Rayes et al. using the same combination supported these findings. A subsequent phase III trial (CanStem111P; NCT02993731) was performed to investigate napabucasin for the treatment of 1134 metastatic PDAC patients combined with nab-paclitaxel and gemcitabine [102]. However, the trial was terminated in 2019 due to 50% futility observed at interim analysis [103]. Regardless, another phase III study employing napabucasin in combination with weekly paclitaxel and low-dose gemcitabine in 230 patients with metastatic pancreatic cancer is ongoing (NCT03721744).

#### 6.1.3. Src Inhibition

Inhibition of the non-RTK Src may be an effective treatment option due to its overexpression in pancreatic cancer. However, up to date, clinical trials using Src inhibitors in solid tumours have not shown promising results, with little to no benefits seen in small cell lung cancer and metastatic colorectal cancer, particularly [104]. Further clinical investigations are ongoing, including clinical trials for the treatment of pancreatic cancer.

Dasatinib is a competitive inhibitor of Src. The use of dasatinib as a monotherapy has failed to demonstrate clinical benefit in patients with metastatic PDAC following a phase II study (NCT00474812) [105]. This was due to drug resistance mechanisms mediating upregulation of alternate signalling pathways including the PI3K/AKT and MAPK pathways in response to dasatinib treatment [106]. However, promising results have been observed following combination therapy. For example, one phase I clinical trial treating 47 patients with advanced solid tumours with dasatinib plus gemcitabine showed encouraging results for pancreatic cancer inducing stable disease in two out of eight patients with gemcitabine-refractory PDAC (NCT00429234) [107]. This led to the initiation of a subsequent phase II study where 202 patients with locally advanced PDAC were treated with the same combination of dasatinib plus gemcitabine or gemcitabine plus placebo. However, results did not show statistically significant difference in OS or PFS between the two treatment groups (NCT01395017) [108]. In addition, toxicities were found to be higher in the dasatinib-treated cohort compared to the placebo group.

Dual Src/EGFR inhibition has also been proposed as a treatment option for pancreatic cancer following a promising preclinical study [109]. This was explored in a phase I clinical trial whereby 19 patients with advanced pancreatic cancer were treated with dasatinib plus erlotinib (an EGFR inhibitor) (NCT01660971) [110]. Stable disease was induced in 69% of patients, although several grade 1/2 toxicities were observed. Interestingly, Dosch et al. have recently demonstrated that dasatinib plus erlotinib inhibits STAT3 whilst enhancing micro-vessel density and inducing stromal remodelling in PDAC tumours using a Ptf1a^cre/+^; LSL-Kras^G12D/+^; Tgfbr2^flox/flox^ transgenic mouse model [111].

Clinical trials exploring the efficacy of dasatinib for the treatment of pancreatic cancer are still ongoing (see Table 6). One phase II study is currently treating 44 metastatic PDAC patients with dasatinib plus mFOLFOX6 (oxaliplatin, leucovorin and 5-FU) (NCT01652976). The phase II MATCH screening trial also involves dasatinib for the treatment of patients harbouring discoidin death receptor 2 (DDR2) mutations—specifically S768R, I638F, or L239R (NCT02465060). However, these mutations are not commonly observed in pancreatic cancer (approximately 0.57% of patients).

### 6.2. MAPK/ERK Pathway

Initiation of the mitogen-activated protein kinase/extracellular signal-regulated kinase (MAPK/ERK) signalling pathway occurs following extracellular ligand binding to RTKs. One of the most studied pathway activators is the epidermal growth factor (EGF) which binds to its corresponding receptor, EGFR. This initiates the recruitment of Son of Sevenless (SOS), a guanine nucleotide exchange factor that converts Ras to its activated form. As a result, Raf is recruited to the plasma membrane, followed by the mitogen-activated protein kinase kinase (MEK1/2), leading to ERK1/2 activation [112]. This initiates ERK1/2 translocation to the nucleus activating several downstream targets mediating cell survival, proliferation, differentiation and inflammation [113].

**Figure 5:**
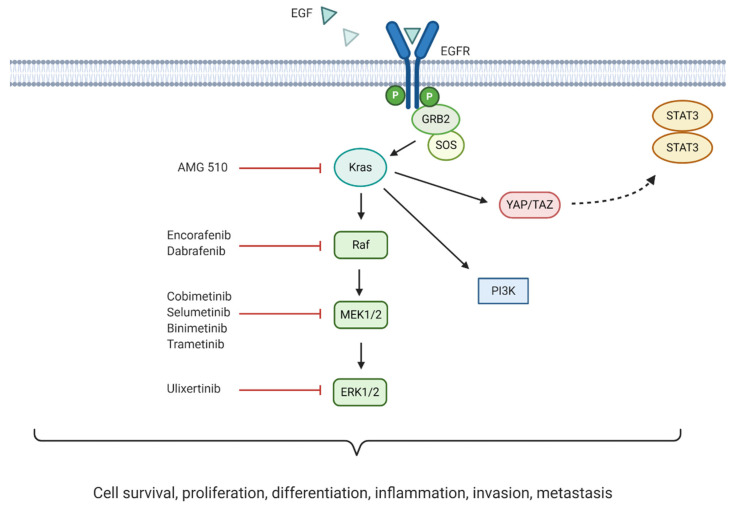
Mitogen-activated protein kinase (MAPK)/ extracellular signal-regulated kinase (ERK) pathway crosstalk, downstream effects, and inhibitors currently being trialled in pancreatic cancer treatment.

MAPK/ERK signalling requires tight control to maintain cellular homeostasis. Importantly, as MAPK/ERK pathway dysregulation is common in human cancers, with several pathway components acting as oncogenes, different therapeutic molecular targets are being explored [113,114]. Overall, the significance of MAPK pathway dysregulation in pancreatic cancer development and progression indicates that its targeting may lead to better patient outcomes. A summary of the pathway and corresponding inhibitors currently undergoing clinical trials for the treatment of pancreatic cancer can be seen in Figure 5.

#### 6.2.1. KRAS Inhibition

Mutations in the KRAS oncogene have been associated with one third of all cancers. Remarkably, 90% of all pancreatic cancers harbour this mutation [115]. The constitutive activation of the encoded mutant Kras protein mediates continuous downstream MAPK signalling. Moreover, mutant Kras has been associated with direct activation of the PI3K and YAP/TAZ signalling pathways, with the latter thought to activate the JAK-STAT3 pathway [116,117]. The most commonly observed KRAS mutations in PDAC are G12D and G12V (51% and 30%, respectively), although mutations in G12C do occur at a lower rate of 2% [118]. Inhibition of Kras downstream effectors (Raf, MEK, and ERK) has been explored. However, the use of these inhibitors has proven ineffective due to compensatory PI3K pathway reactivation, and dual targeting of these pathways presents with severe toxicity [118].

Recently, the discovery of AMG 510 (sotorasib)—an inhibitor targeting KRAS G12C—has produced promising results in preclinical studies and early clinical trials (NCT03600883). Initial results demonstrated partial responses in two of four NSCLC patients, with stable disease achieved in the remaining two [119]. These encouraging findings led AMGEN to initiate a larger scale phase I/II study enrolling 533 patients with advanced/metastatic solid tumours harbouring KRAS G12C mutations (NCT03600883) [120]. As of January 2020, early results appear promising following treatment of 129 patients (59 NSCLC, 42 colorectal cancer, and 28 others, including pancreatic cancer). Six out of eight evaluable pancreatic cancer patients achieved stable disease, with three showing reduction in tumour burden of roughly 30% from baseline.

Limitations associated with the use of AMG 510 have been identified in preclinical studies via accumulation of active EGFR as a compensatory growth mechanism following treatment [118,119]. This suggests that combining AMG 510 with EGFR inhibitors may bypass this potential early adaptive resistance mechanism. Other KRAS inhibitors have also been discovered, including MRTX849 (a selective KRAS G12C inhibitor) and compound 11 (a wild-type plus mutant KRAS inhibitor) [121]. Clinical trials using MRTX849 in advanced/metastatic cancer harbouring KRAS G12C mutations are currently ongoing (NCT03785249 and NCT04330664). Although KRAS G12C mutations are not as frequent in pancreatic cancer, these results collectively provide hope for the development of other similar therapeutics targeting the more commonly observed KRAS G12D and G12V mutations.

#### 6.2.2. BRAF Inhibition

The existence of a subset of BRAF-mutant pancreatic tumours encoding constitutively active mutant B-Raf, 3% of pancreatic tumours, has led to the development of targeted therapeutics. All three BRAF inhibitors currently in clinical phase—encorafenib, vemurafenib, and dabrafenib—have been approved to treat BRAF-mutant advanced melanoma. Promising results have led to their evaluation for pancreatic cancer. Encorafenib (Braftovi) is considered better than dabrafenib and vemurafenib due to its 30 h half-life, which reduces adverse events commonly associated with BRAF inhibition [122]. A phase Ib/II study analysed the effects of encorafenib in combination with the MEK inhibitor binimetinib to treat patients with BRAF V600E-mutant solid tumours. Findings showed this combination to be better tolerated than other BRAF/MEK combinations (NCT01543698) [123]. At present, one phase II clinical trial is exploring the effectiveness of encorafenib in combination with binimetinib for the treatment of BRAF-mutant pancreatic tumours (NCT04390243). Dabrafenib (Tafinlar) has also shown promising results in pancreatic cancer [118,119,124]. One case study presented a 49-year-old female with metastatic pancreatic cancer (harbouring BRAF and P53 mutations) responding to gemcitabine followed by dabrafenib and trametinib (a MEK inhibitor) treatment [120]. Dabrafenib is currently on the list of therapeutics being used in the phase II MATCH trial (NCT02465060).

#### 6.2.3. MEK Inhibition

MEK1 and MEK2 are very closely related kinases that have demonstrated functional differences in pancreatic cancer cells (PC-1.0 cells), with MEK1 being linked to cell proliferation and MEK2 to invasive capacity. Inhibition of MEK to prevent the downstream activation of ERK1/2 has been extensively explored [112]. To date, MEK inhibitors have generally failed to improve OS in pancreatic cancer when used as a monotherapy or in combination with chemotherapy. This is likely due to toxicities associated with MEK inhibition, but could also be due to therapeutic resistance whereby downstream ERK is reactivated [121,125]. Therefore, simultaneous targeting of multiple pathway components may prove beneficial in the treatment of KRAS-mutant pancreatic tumours, if toxicities remain manageable.

The MEK1/2 inhibitor cobimetinib (Cotellic), has been approved for the treatment of a subset of unresectable/metastatic melanoma and is currently being explored in phase I/II clinical trials for PDAC treatment [126]. A clinical trial has tested cobimetinib in combination with gemcitabine to treat patients harbouring KRAS G12R mutant pancreatic tumours with two previously failed standard chemotherapeutic treatments. Although the sample size was small and results were only observed in this specific subset of KRAS mutant PDAC cases, five out of six patients enrolled achieved stable disease, with one partial response observed [127]. Cobimetinib is currently being explored alongside the use of other targeted therapies in phase I/II clinical trials (see Table 7). For example, one phase I/II study is evaluating immunotherapy-based treatment combinations in PDAC patients (NCT03193190), and another one the combination of cobimetinib with RMC-4630 (another MAPK pathway inhibitor, acting via inhibition of the SHP2 oncogene) [128] in patients with solid tumours, including PDAC cases (NCT03989115).

Selumetinib (Koselugo; AZD6244; ARRY-142886) is a selective MEK1/2 inhibitor that has been trialled as a therapeutic option for pancreatic cancer. One randomised phase II study compared its effectiveness against capecitabine in 70 patients with advanced/metastatic pancreatic cancer, previously treated with gemcitabine (NCT00372944) [125]. No significant differences in OS were observed between the two treatment groups, although selumetinib was found to be safe and well-tolerated. Combination therapy has subsequently been explored, with a phase II study investigating the efficacy of selumetinib and erlotinib (an EGFR inhibitor) for the treatment of advanced, previously chemotherapy-refractory PDAC (NCT01222689) [129]. Results were promising, showing prolonged disease control and evidence of anti-tumour activity in 19 out of 49 patients. Selumetinib is also being tested for patients with locally advanced/metastatic KRAS G12R mutant pancreatic cancer (NCT03040986).

The safety of another MEK1/2 inhibitor, binimetinib (MEK162; ARRY-438162), is currently being evaluated in phase I/II clinical trials in combination with other therapeutics (see Table 7). One study is exploring the effect of combining binimetinib with avelumab/talazoparib (PD-L1/PARP inhibitor, respectively) for the treatment of locally advanced/metastatic pancreatic cancer (NCT03637491). The trial NCT04390243 is looking at binimetinib combined with encorafenib (a BRAF inhibitor) for the treatment of pancreatic cancer patients with somatic BRAF V600E mutations. Binimetinib is also being explored in the phase II MATCH trial.

Trametinib (GSK1120212) has received FDA approval as a monotherapy for the treatment of unresectable/metastatic BRAF-mutant malignant melanoma and for the treatment of anaplastic thyroid cancer in combination with dabrafenib (a BRAF inhibitor). However, less promising results have been observed in pancreatic cancer patients [114,130]. One phase II clinical trial using trametinib alongside gemcitabine failed to show significant improvements in OS, PFS, ORR, and duration of response compared to gemcitabine alone, in 160 patients with previously untreated metastatic pancreatic cancer, regardless of their KRAS mutation status (NCT01231581) [121]. Similarly, another phase II clinical study using trametinib in combination with the focal adhesion kinase (FAK) inhibitor, GSK2256098, showed no clinical activity in 11 advanced PDAC patients, although correlative studies to investigate RNA-expression subtypes and pathway inhibition markers are ongoing (NCT02428270) [131]. At present, trametinib is being assessed in the phase II MATCH trial (NCT02465060).

#### 6.2.4. ERK Inhibition

ERK1 and ERK2 are ubiquitously expressed, functionally redundant kinases forming part of the MAPK/ERK pathway. Although inhibition of ERK may prevent the aforementioned resistance associated with inhibition of upstream MAPK pathway elements, it has also been linked to elevated levels of autophagy and PI3K upregulation in PDAC [132,133]. Combining ERK inhibition with autophagy/PI3K inhibitors may therefore enhance therapeutic effectiveness. Ulixertinib (BVD-523) inhibits both ERK1 and 2 and has shown promising results for the treatment of advanced solid tumours. One phase I clinical trial observed partial responses in 14% of patients with advanced solid tumours that completed two ulixertinib treatment cycles, although the number of pancreatic cancer cases included in this percentage is unclear (NCT01781429) [134]. A recent phase I clinical trial using ulixertinib in combination with nab-paclitaxel and gemcitabine for the treatment of 18 patients with metastatic pancreatic cancer was terminated due to adverse events (NCT02608229). This therapeutic is however currently being explored in the phase II MATCH trial (NCT02465060).

### 6.3. PI3K/AKT/mTOR Pathway

**Figure 6:**
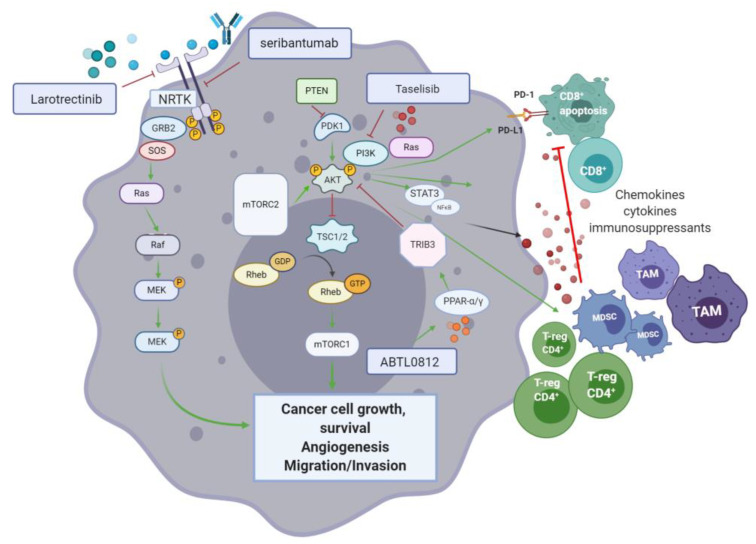
The PI3K/AKT/mTOR pathway in pancreatic cancer is central for cancer cell growth, survival, migration, invasion, angiogenesis, and modulation of the anti-cancer immune response. Inhibition of different elements of this signalling pathway is shown to reduce disease progression and promote CD8^+^ T-cell infiltration in pancreatic ductal adenocarcinoma (PDAC).

The role of the phosphoinositide 3-kinase (PI3K), AKT (also known as protein kinase B) and mammalian target of rapamycin (mTOR) pathway in cancer, is well established [135]. The PI3K/AKT/mTOR pathway (Figure 6) controls molecular processes such as cell cycle, metabolic homeostasis, angiogenesis, proliferation, differentiation, and survival. PI3Ks are lipid kinases that upon phosphorylation by specific kinases (i.e., RTK and RAS), relocate to the cellular plasma membrane, and integrate signals from growth factors, chemokines/cytokines and extracellular matrix processes, through PI3Ks mediated downstream signalling [136]. Depending on substrate specificity, PI3Ks can activate AKT or mTORC2 (a downstream target of AKT), regulating cellular proliferation and metabolism. When aberrant activation of upstream mutated tyrosine kinase receptors (TKRs) and RAS, as well as, functional loss of the tumour suppressor gene PTEN (phosphatase and tensin homolog) and INPP4B (type II inositol polyphosphate-4-phosphatase) occur, the PI3K/AKT/mTOR pathway becomes constitutively activated—A hallmark of many cancers, including pancreatic [136].

With respect to the role of the PI3K/AKT/mTOR pathway in cancer immune evasion, a link with the programmed death-1/programmed death-ligand 1 (PD-1/PD-L1) pathway is suggestive of an immunomodulatory role of the former. PI3K activation impairs tumour infiltration by CD8^+^ T-cells inducing their apoptosis through the increased expression of PD-L1 on the surface of cancer cells. Blocking agents targeting the PD-1/PD-L1 pathway also disrupt the PI3K/AKT/mTOR pathway, resulting in inhibition of pancreatic cancer growth, progression and metastatic potential, as observed in an orthotopic mouse model of PDAC. Moreover, the release of chemokines and cytokines by tumour-associated immunosuppressive cells is promoted by PI3K/AKT/mTOR [136]. Reciprocally, inhibition of the PI3K/AKT/mTOR pathway has shown to control CD4^+^ regulatory T-cells activity and enhance cancer cell recognition by the immune system in vivo [137]. AKT is pivotal for the regulation of metabolic processes that are augmented in cancer cells. AKT also promotes disease progression through disruption of CD8^+^ T-cell mediated apoptosis and the enhancement of immunosuppressive regulatory T-cells. The target of rapamycin (mTOR) is a key Ser/Thr kinase of the PI3K signalling cascade. Composed of two subunits (mTORC1 and mTORC2), the mTOR complex jointly acts to promote nucleotide, protein, and lipid synthesis (mTORC1), as well as phosphorylation of AGC kinases (PKA, PKG, and PKC). In PDAC, the phosphorylation of Ser2448 in mTOR results in activation of the MEK/ERK and PI3K pathways. Therefore, mTOR is a relevant target for the treatment of PDAC [138].

#### 6.3.1. Surface RTK Blockade Targeting for PIK/AKT/mTOR Inhibition

Seribantumab (MM-121; Sanofi/Merrimack pharmaceuticals) is a humanised monoclonal antibody that targets ErbB3—EGF receptor 3 kinase (HER3). In 2017, seribantumab was FDA approved for the treatment of non-small cell lung (NSCLC) cancer. HER3 is a key activator of PI3K/AKT and its inhibition has recently been studied alone and in combination with other agents (cetuximab, irinotecan, and paclitaxel) for the treatment of several cancers including NSCLC, breast, colon, head and neck, and ovarian. An open label, multi-centre (USA) phase II trial is evaluating seribantumab for patients with locally advanced, metastatic solid tumours, including PDAC cases, harbouring the neuregulin1 (NRG1; a HER3 ligand) fusion gene (NCT04383210) (see Table 8).

Fusions of neurotrophic receptor tyrosine kinase 1-3 (NRTK1-3) genes result in constitutive activation of RTKs (A-C) and have been described as driver mutations in pancreatic cancer as well as in various other solid tumours [139]. In this context, larotrectinib (Vitrakvi; Loxo Oncology) is a small molecule, competitive inhibitor of RTK A-C, which received an orphan drug designation (soft tissue sarcomas, 2015) and breakthrough therapy designation for treating metastatic, NRTK fusion positive solid tumour in 2016, and was approved by the FDA in 2018. In a phase I/II trial which included 55 patients with RTK fusion positive solid tumours (including pancreatic cancer, among others), treatment with larotrectinib demonstrated an ORR of >75% (95% CI, 61–85) [140]. A more recent publication by Hong et al. summarising findings from three phase I/II trials using larotrectinib, reported an objective response of 79% (95% CI, 72–85) out of 159 patients, while the drug was well tolerated and its long-term administration was feasible. As part of the phase II MATCH screening trial (NCT02465060), larotrectinib will be tested in NRTK fusion positive pancreatic cancer patients.

#### 6.3.2. Small Molecules and Novel Inhibitors of the PI3K/AKT/mTOR Pathway

PI3K has been widely studied as an attractive target for therapies disrupting the PI3K/AKT/mTOR axis [136]. The small molecule taselisib (Roche) is an isoform-selective PI3K inhibitor which allows the targeting of mutant PIK3CA cancers. In a phase I basket study enrolling 166 patients with PIK3CA mutant solid tumours, taselisib showed limited activity (9% RR), with a partial response in a subset of pancreatic cancer patients (sarcomatoid and pseudopapillary). Based on these results, the development of taselisib was halted [141]. ABTL0812 is a small molecule that inhibits AKT phosphorylation via upregulation of Tribble3 (TRIB3)—A pseudokinase inhibitor of AKT. ABTL0812 targets the peroxisome proliferator-activated receptors (PPARs) α and γ resulting in upregulation of TRIB3 expression. A phase I (open label) followed by a phase II randomised study combining ABTL0812 with FOLFIRINOX, as first line treatment in metastatic pancreatic cancer, is currently underway (NCT04431258). Another phase I/II trial (NCT03417921) using ABTL0812 alongside gemcitabine and nab-paclitaxel, in a similar cohort, has been registered.

## 7. Immunotherapy

**Figure 7:**
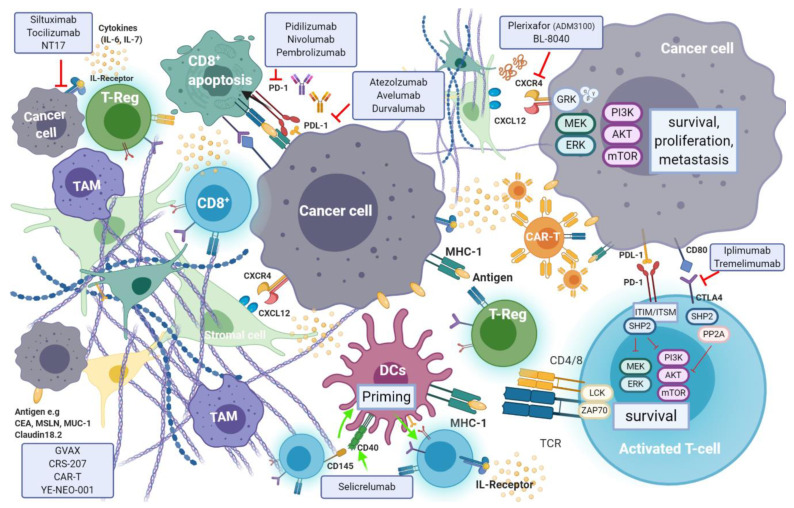
Cancer cell mediated activation of immunosuppressive cells is promoted through secretion and expression of immunosuppressive cytokines and membrane bound ligands (e.g., PDL-1, CTLA-4), that further hinder anti-tumour response. Regulation of T-cell response occurs through T-cell receptor (TCRs) interaction with their respective ligands, following which signalling cascade action antagonizes T-cell activation. The induction of endogenous cancer cell survival, proliferation and metastasis by pancreatic stellate cells (PSCs) is enabled through the CXCR4-CXCL12 axis. Such processes are targeted in immunotherapy with the aim of triggering anti-tumour immunity or direct targeting of cancer cells.

Recent evidence suggests that efficient immune response directed against tumour-specific neoantigens is often dampened by an immunosuppressant TME, which is a hallmark of pancreatic cancer [9]. Cancer cell mediated activation of immunosuppressive cells such as myeloid-derived suppressor cells (MDSCs), tumour associated macrophages (TAMs) and CD4^+^ T-regulatory cells (T-reg), is promoted through secretion and expression of immunosuppressive cytokines and membrane bound ligands (e.g., Programmed Death Ligand-1(PDL-1) or B7-1/2), that further hinder natural killer (NK) and T-cell anti-tumour response [142]. Regulation of T-cell response occurs through T-cell receptor (TCRs) interaction with their respective ligands, following which signaling cascade action antagonizes T-cell activation [143]. The induction of endogenous CD8^+^ T-cell reaction is blunted by pancreatic stellate cells (PSCs) which also promote T-reg and MDSC differentiation (Figure 7).

Moreover, a population of immature and poorly functioning antigen-presenting cells (APC) dendritic cells (DCs), that would normally induce anti-tumour immune response, are characteristic elements in pancreatic cancer. The relatively low mutational burden in pancreatic cancer, as opposed to other tumours, results in lower expression of tumour neo-antigens and therefore a reduced susceptibility to immune-surveillance [144]. In pancreatic cancer, longer survival is observed in patients with tumours that are efficiently infiltrated by CD8^+^ T-cells, FoxP3^+^ and NK cells, and carry higher volumes of neo-antigens [145]. A higher density of CD4^+^ T-regulatory and T_H_2-helper cells, paucity of CD8^+^ T-cells and lower mutational burden are associated with impaired anti-tumour immunity and shorter survival rates. Targeted therapies seek to enhance immune effector cell response in cancer. T-cell immune responses are regulated by mechanisms known as “immune checkpoints”. Tumours exploit checkpoint mediated inhibition of cytotoxic T-cells to evade endogenous anti-tumour immune response. Targeted inhibition of specific immune checkpoints aims to disrupt this evasion mechanism and enhance tumour infiltration by cytotoxic immune effector cells. The two most frequently studied checkpoints are PD-1 and its ligand—programmed death-ligand 1 (PDL-1), as well as the cytotoxic T lymphocyte protein-4 (CTLA-4). Several antibodies targeting these checkpoints are being investigated, while others have already been approved for use.

### 7.1. Immune Checkpoint Inhibitors in Pancreatic Cancer

#### 7.1.1. Targeting PD-1 and PD-L1

Programmed cell death protein 1 (PD-1) is an inhibitory receptor expressed on the surface of a variety of immune cells including T-cells, monocytes and B-cells. PD-1 is a crucial immune checkpoint molecule that inhibits the function of CD4^+^ and CD8^+^ T-cells in the TME and regulates its function during different physiological responses such as cancer, autoimmunity, and infection. The main PD-1 ligands are PD-L1 and PD-L2, which can lead to inhibitory cell singling resulting in the suppression of T-cell proliferation and cytokine production leading to the inhibition of mediated T-cell immune response [146] (see Figure 6). PD-L1 is expressed on the surface of many cell types, including APC, T and B cells, monocytes, and epithelial cells. Moreover, it is upregulated in response to proinflammatory cytokines such as IFNγ, IL-4, STAT1, and IFN regulatory factor-1 (IRF1). In addition, PD-L1 upregulation has been described in different types of cancers [147]. In contrast to PD-L1 expression, PD-L2 expression is limited mainly to APC cells.

The overexpression of PD-L1 in cancer cells allows them to escape T-cell mediated immune response [148]. Blocking the interaction between PD-1 and its ligand PD-L1 improves T-cell function leading to cancer cell immune recognition [149]. Therefore, this particular interaction has gained much popularity as a therapeutic target for the treatment of several cancers, including pancreatic cancer, as it has been linked to poor prognosis. The KEYNOTE-028 trial (NCT02054806) evaluated the effect of the PD-1 antagonist pembrolizumab (Keytruda^®^) in a cohort of 20 patients with various advanced solid tumours [150]. In the subset population with pancreatic cancer, the use of pembrolizumab in monotherapy resulted in lack of efficient ORR (0–14%) and PFS of 1.7 months (95% CI, 1.5–2.9 months).

Resistance mechanisms are attributed to the often low mutational burden, the high population of suppressant immune cell population and CD8^+^ T-cell sequestration. This has inspired a shift toward a multi-faceted approach, utilising combinations of immunomodulatory agents in an attempt to simultaneously target several aspects of drug resistance in PDAC. However, boosted PD-L1 expression does not necessarily sensitise PDAC to anti PD-1/PD-L1 therapies. In contrast to vaccine therapy, where it induced infiltration of PD-1 effector T-cells, chemo- or radio-therapy did not have the same effect. As a result, T-cell inducing agents may be crucial for the combination of chemo- or radio-therapy and immune checkpoint inhibitors, to improve the outcome of PDAC patients. A phase I/II trial completed in 2018 (NCT02331251), reported the use of gemcitabine, nab-paclitaxel and pembrolizumab (arm 3) as safe for first line treatment in PDAC. However, treatment efficacy reported only faintly improved outcomes compared to outcomes with a 28-day course, three treatment cycle, with gemcitabine and nab-paclitaxel [151].

An ongoing randomised phase II study (NCT03727880) is looking into the effectiveness and safety of combining standard pancreatic cancer chemotherapy, prior and post-surgery, and pembrolizumab with or without the FAK inhibitor defactinib, in patients with high-risk resectable pancreatic cancer. The aim is to investigate whether reprograming the tumour microenvironment (FAK targeting) followed by chemotherapy can improve the effect of pembrolizumab. FAK inhibition by defactinib, could block the activation of downstream signalling pathways such as RAS/MEK/ERK and PI3K, inhibiting tumour proliferation and migration. Defactinib is also included in the phase II MATCH trial.

A 2-armed randomised phase III study (NCT03983057) is currently comparing the effect of modified-FOLFIRINOX alone in combination with the anti-PD-1 monoclonal antibody nivolumab, in patients with borderline resectable and locally advanced pancreatic cancer. The safety and efficacy of the co-administration of nivolumab and ipilimumab (anti-CTLA-4) together with high dose radiation therapy, in patients with metastatic pancreatic cancer, biliary tract cancer or in patients with previous gemcitabine intolerance, will be assessed in another phase II randomised open label trial (NCT02866383). Results are expected towards the end of 2021. A phase I/II trial (NCT03767582) is investigating the safety of nivolumab in combination with BMS-813160 (a CCR2/CCR5 dual antagonist) and GVAX (pancreatic cancer vaccine) in locally advanced pancreatic cancer patients. Those patients would have already received chemotherapy and radiotherapy, but the study aims to evaluate whether this combination therapy enhances CD8^+^ CD137^+^ cells infiltration in PDAC.

#### 7.1.2. Targeting the Cytotoxic T-Lymphocyte Associated Antigen-4 (CTLA-4)

Activated T-cells express the cell surface receptor CTLA-4 (cluster of differentiation (CD)152), which upon binding to its activating ligands, B7-1 (CD80) and B7-2 (CD86), triggers a signal cascade that results in T-cell inactivation and apoptosis. Impairment of T-cell mediated anti-tumoural immune response therefore follows. An inverse correlation between expression levels of B7 by cancer cells (and tumour infiltrating monocytes, APCs and T-cells) and survival exists, which varies between different tumour types [152]. In PDAC, expression of B7 ligand by tumour cells is upregulated as the disease progresses.

The only anti CTLA-4 blocking monoclonal antibody approved for clinical use is a direct antagonist of CTLA-4—iplimumab (YERVOY^®^, Bristol-Mayers Squibb, New York, NY, USA). Promising preclinical studies formed basis for its regulatory approval for the treatment of advanced stage malignant melanoma in 2011 [153], and is currently being investigated in other cancers such as NSCLC, advanced prostate and bladder cancers. Three phase Ib dose escalation studies (NCT01473940, NCT01473940 and NCT01473940) of iplimumab and gemcitabine in patients with late stage (III and IV) pancreatic cancer, showed minimal benefit over gemcitabine alone (RECIST study; ORR 14, 15% and stable disease in seven, five, and five patients, respectively). In a phase II study of iplimumab as a single agent in locally advanced or metastatic PDAC, no response was observed apart from one delayed response after initial disease progression (Table 9). The one patient with a delayed response was reported to have a reduction in both primary and metastatic lesion tumour burden [154].

Tremelimumab (CP-675 206) is a CTLA-4 specific fully humanised monoclonal antibody. Tremelimumab has been investigated in various cancers including gastric, pancreatic, mesothelioma, renal, melanoma and breast [143]. In a dose escalation study (NCT00556023) in 34 patients with metastatic PDAC, the combined administration of gemcitabine and tremelimumab led to a partial response in only two patients (OR of 7.5 months (95% CI, 5.6–9.4 months)). This observation was reported in the cohort of patients subjected to the highest tremelimumab dose studied (15 mg/Kg). A phase II study of tremelimumab (NCT02527434) as monotherapy however, reported poor efficacy in a metastatic disease cohort of patients with previous treatment with 5-FU or gemcitabine-based chemotherapy. Ninety percent of patients (18/20) had disease progression with a poor median OS of 4 months (95% CI, 5.8–9.4 months) [155].

### 7.2. The CXCL12-CXCR4 Axis

CXCR4 is a pan-cancer G-coupled receptor expressed in both solid and haematological malignancies. Cancer stromal cells in PDAC abundantly express C-X-C Motif Ligand 12 (CXCL12)—A ligand of CXCR4. CXCL12-CXR4 axis activation results in tumour progression, survival, enhanced invasion and metastasis, as well as TME modulation [156]. Through increased expression of this ligand in metastatic sites of pancreatic cancer, CXCL12 is also thought to have a cancer cell-homing function at the target organs. In more aggressive subtypes, invasive CXCR4^+^ cancer stem cells have also been identified at distant sites, suggestive of the CXCL12-CXCR4 axis role in invasion and metastasis [157]. The role of CXCL12-mediated activation of FAK, ERK and AKT, has also been associated with chemoresistance in pancreatic cancer, among other gastrointestinal cancers [157]. PDAC is described as a “cold” tumour in which an imbalance between immunosuppressive populations of CD4^+^ T-cells and cytotoxic CD8^+^ exists. Chemotaxis of immunosuppressive cells (MDSCs, T-Regs, PDL-1+ mast cells) and CD8^+^ T-cell sequestration is enabled through the activation of the CXCL12-CXCR4 axis, and its interruption results in higher CD8^+^ T-cell infiltration and intra-tumoural density, especially when used in combination with immune checkpoint inhibitors.

Plerixafor (AMD3100; Mozobil; Sanofi Genzyme, Cambridge, MA, USA) is the only FDA approved CXRC4 inhibitor. It has been widely studied in the context of treatment of acute myeloid leukemia, and in preclinical mouse models of PDAC [158,159]. Encouraging preclinical findings formed basis for clinical studies of plerixafor in patients with advanced PDAC, with a phase I (NCT02179970) and an open label phase II (NCT04177810) studies currently underway. The latter will assess the response of plerixafor in combination with the anti PD-1 antibody cemiplimab (REG-2810; Regeneron) in metastatic PDAC patients and is expected to report findings in late 2024.

BL-8040 (Motixafortide; BiolineRX, Modiin, Israel) is a high affinity, synthetic inhibitory peptide of CXCR4 which promotes bone marrow and lymph-node lymphocyte and NK cell mobilisation alongside inhibition of T-reg cell function in various solid tumours. Moreover, a synergistic effect was observed when BL-8040 was used in combination with PD-1 blockade, in preclinical models of PDAC. In a single arm phase IIa study (the COMBAT study; NCT02826486) the efficacy of BL-8040 together with pembrolizumab (keytruda^®^, Merk, Kenilworth, NJ, USA) as second or third line therapy in pancreatic cancer (cohort 1, *n* = 37), was compared with a second cohort receiving both agents together with chemotherapy (the NAPOLI-1 regimen: liposomal irinotecan, fluorouracil, and leucovorin) (cohort 2, *n* = 16). The highest benefit was observed when used in a second line setting, with the triple-therapy combination showing an ORR of 32% and a disease control rate of 77%. A median duration of response of 7.8 months was recorded in this cohort. The COMBAT/KEYNOTE-202 trial (NCT02826486) is assessing BL-8040 in combination with pembrolizumab in patients with metastatic pancreatic cancer. BL-8040 is also being evaluated in a phase I/II trial investigating multiple combinations of immunotherapy agents in treatment naïve, and in those with one-line prior systemic therapy (NCT03193190).

### 7.3. The Role of CD40, Immunocytokine and the Adenosine Pathway in PDAC Immunotherapy

CD40—A member of the tumour necrosis factor receptor superfamily, is a surface marker expressed by B-cells, antigen presenting DCs, monocytes as well as various cancer cells [160]. The activation of CD40 occurs through T-cell presentation of its ligand CD154, by triggering downstream signalling cascades that result in the priming of CD40 expressing DCs. These in turn further induce the activation of cytotoxic T-cells and the release of pro-inflammatory cytokines, as part of cell mediated immune response. Cytokines (e.g., G-CSF, IL-2, IL-21, and IL-15) can be harnessed to stimulate an anti-tumour immune response by activation of DCs, NK and effector T-cells in combination with immune checkpoint inhibition, to transform the immunosuppressant TME of PDAC into an inflammatory one in vivo [161]. Clinical studies of CD40 and cytokine-mediated therapy have been less successful. Selective activation of CD40 using the fully human monoclonal antibody selicrelumab, as single agent, showed limited clinical efficacy in 27 patients with advanced solid tumours [162]. A more recent phase Ib study (NCT02760797) similarly reported lack of objective clinical response to CD40-targeted activation with selicrelumab, not even when used in combination with CSF-1R blockade (emactuzumab) [163].

Several pro-inflammatory cytokines have been associated with disease progression, chemoresistance and poor outcomes in pancreatic cancer. In particular, IL-6 is currently being studied as a potential target for anti-inflammatory therapy. Siltuximab and tocilizumab are IL-6 targeting mAbs, which are FDA approved for treatment of several rheumatic, lymphoproliferative, and cytokine release syndromes. They have also been tested in various solid tumours (renal, ovarian, and multiple-myeloma) [164]. The effects of IL-6 blockade in combination with other checkpoint inhibitors (e.g., anti PD-1 and anti CTLA-4) is currently being evaluated in phase I/II trials in metastatic pancreatic cancer (NCT04191421 and NCT04258150). The benefit of using tocilizumab in combination with gemcitabine and nab-paclitaxel is also being assessed in a randomised multi-national phase II study (PACTO; NCT02767557) in 140 patients, as first line treatment in locally advanced or metastatic PDAC. IL-7 plays a part in lymphocyte (T-cell and B-cell) development and homeostasis and therefore, its dysregulation supports autoimmunity and carcinogenesis. Overexpression of IL-7R in pancreatic cancer strongly correlated with disease progression following tumour resection, as well as with low survival rates [165]. The effects of NT17 (efineptakin-α)—a long acting IgG fusion (recombinant IL-7) protein, is currently being tested in advanced, relapsed and refractory to previous treatment solid tumours (breast, lung, colorectal and pancreatic—or any other advanced solid tumours). This phase Ib/IIa study (KEYNOTE A60, NCT04332653) will also assess the effect of combining immunotherapy with pembrolizumab (anti PD-1). Results from the KEYNOTE A60 study are expected in 2023.

The adenosine pathway has recently been studied as a novel strategy to enhance anti-tumour immune response in cancers, due to its key role in immunosuppression, angiogenesis and ischaemic preconditioning. Activation of adenosine-mediated immunomodulation interestingly, has shown to be both of immunosuppressant and stimulant nature [166]. Extracellular levels of ATP peak with rising cellular stress associated with inflammation, hypoxia and necrosis in the TME. Adenosine is the end product of ATP degradation by specific ecto-nucleosidases in both canonical (CD73, CD39) and non-canonical mediated pathways (CD38 and CD203a). Through the former, ATP is degraded into AMP by CD39 and later dephosphorylated to form adenosine by CD73. CD39 expression on T-regulatory cells, DCs, and macrophages is induced in a hypoxic environment. CD73 is expressed on tumour and immunosuppressant cells such as MDSCs and T-reg cells [166]. By binding specific purinergic receptors (A2sR and A2bR), adenosine induces an immunosuppressive downstream signalling cascade as a consequence of intracellular cAMP accumulation. CD39 independent (non-canonical) adenosine production is enabled through NAD^+^ conversion into AMP by CD38 (NAD^+^ nucleosidase) and CD203a (ecto-nucleotide pyrophosphatase/phosphodiesterase 1), which is complemented by CD73 mediated AMP to adenosine conversion [166]. With respect to pancreatic cancer, adenosine signalling plays a key role in pancreatic exocrine physiology, and together with CD73 and CD39, has been extensively reported to be upregulated in PDAC and its TME, in preclinical work [167]. Interestingly however, in a murine model (BxPC-3-GFP cells in BALB/c mice) of pancreatic cancer, adenosine was shown to also have anti-tumourigenic effects by inducing cancer cell apoptosis. This effect, which was linked with structural similarities between adenosine and gemcitabine, was further augmented by inhibition of the AKT/p21 axis using the Akt inhibitor GSK690693 (GlaxoSmithKline, London, UK) [168]. The evaluation of adenosine pathway targeting in clinical trials is however in its early stages and active clinical trials are limited to phase I or phase I/II studies—the majority of which have not yet started subject recruitment.

Oleclumab is a fully human IgG antibody, non-competitive inhibitor of CD73, currently being tested in 20 NCT registered clinical trials for treatment of various solid malignancies. It is currently being evaluated in a phase Ib/II in combination with durvalumab (anti PDL-1) and chemotherapy, for the treatment of 339 patients with metastatic pancreatic cancer. The study is expected to complete in late 2022 (NCT03611556). CD38 inhibition using the anti-CD38 antibody daratumumab (Darzalex^®^, Genmab, Copenhagen, Denmark) together with nivolumab (anti PD-1 IgG4) is being analysed in a phase I/II study of 120 patients with advanced pancreatic, lung and breast cancer (NCT3098550).

The Adenosine A2A receptor (A2aR) is the second most commonly used target. Initially investigated as an anti-Parkinson’s agent, A2aR inhibition is thought to play a role in tumour growth and metastasis inhibition. Effector T-cells increased expression of A2aR in inflammation, is inhibitory to T-cell cytotoxicity and inhibits cytokine release. In NK cells, A2aR activation results in their suppression while in regulatory T-cells, expansion and increased immunosuppressant activity follows [167]. The role of A2aR in pancreatic cancer and its treatment is not yet well established. An open label phase II study (NCT03207867) is evaluating the A2aR antagonist NIR178 in combination with spartalizumab (anti PD-1) in multiple solid tumours (including pancreatic).

The use of immune checkpoint inhibitors has shown promising results in various cancers. In pancreatic cancer, the tumour favouring immune landscape challenges the use of single agent checkpoint inhibition and therefore, a multi-faceted approach is recommended. The Morpheus-Pancreatic cancer trial (NCT03193190) is a phase Ib/II multicentre open label study that will assess multiple combinations of immunotherapy agents in 290 patients with metastatic PDAC.

### 7.4. Cancer Vaccines

Priming of T-cells in order to stimulate an efficient anti tumour immune response can be induced using vaccines [169]. While promising results have been observed in other cancers (cervical, melanoma, renal, and breast), the immunosuppressant TME, the low mutational burden (and therefore the paucity of immunity inducing neoantigens), highlight the need for further research of such an approach in pancreatic cancer [170]. Vaccine based antigen delivery aims to sensitise antigen presenting DCs using the introduction of tumour cells, DNA or specific peptides that are further presented to T-cells, triggering their activation. Activated, antigen specific T-cells are then able to infiltrate the tumour and assert their cytotoxic effects as well as stimulate anti-tumour immunity.

Tissue specificity is crucial when inducing a localised immune reaction. Tissue markers that are differentially expressed in pancreatic cancer (CEA, mucin-1 (MUC-1)), mesothelin, mutated KRAS G12, CA19-9, carcinoembryonic antigen-related cell adhesion molecule 6 (CEACAM6) have been recently studied as potentials vaccine targets [171]. Despite of the abundance of various reports of preclinical studies of anti-cancer vaccines in pancreatic cancer, the use of vaccines in the treatment of pancreatic cancer is an emerging field, and to date no such are approved, with only a handful being investigated in phase II/III clinical trials [169]. In this section we will discuss those which have shown in particular promise in PDAC as well as those that reported promising findings in more advanced clinical studies.

#### 7.4.1. BN-CV301

BN-CV301 is a poxviral based vaccine which contains transgenes that encode for the cancer associated antigens CEA and MUC-1 (an inducer of EMT and chemoresistance) that are both highly expressed in several solid tumours, including PDAC. BN-CV301 is a modified version of PANVAC which previously showed to increase antitumour T-cell (CD8^+^) response against highly expressing MUC-1 and CEA tumours in vivo. These translated into clinical benefit in patients treated for breast, ovarian, colon and appendiceal cancers in terms of survival and response to further treatments. In view of lower safety profiles in patients with atopic dermatitis or who are immune-suppressed, as well as, potential cardiac risks associated with a replication capable smallpox delivery vector, PANVAC was later modified to its currently used attenuated version BN-CV301. Favourable safety profile with no dose limiting toxicities was reported in a dose escalation phase I study (NCT02840994). Antigen specific T-cell generation was observed in most patients with a prolonged stable disease most notably observed in those with KRAS mutated tumours, as well as those treated with immune checkpoint inhibitors following BN-CV301. The pancreatic cancer arm of a phase II study (NCT03376659) will evaluate the effects of combining BN-CV301 together with immune checkpoint inhibition (durvalumab) and capecitabine chemotherapy in a metastatic disease setting (Table 10).

#### 7.4.2. GVAX

GVAX is composed of two human pancreatic cancer cell lines genetically modified to secrete granulocyte-macrophage colony stimulating factor (G-CSF). GVAX stimulates long lasting T-cell infiltration into tumours as well as the activation of myeloid cells. Following limited toxicity and augmentation of anti-tumour immune response observed in 14 patients who underwent resection of PDAC [172], GVAX was further tested in a phase II study in 60 patients with resectable disease (NCT00084383). In this study, GVAX was given together with 5-FU. Improved median overall and disease-free survival were observed (24.8 months (95% CI 21.2–31.6) and 17.3 months (95% CI 14.6–22.8), respectively) alongside induction of mesothelin specific CD8^+^ T-cell response. The benefit of combining GVAX with cyclophosphamide (single injection or multiple doses) versus GVAX alone, is currently under study in a 72-patient cohort—phase II study in resected (or stage I-II) disease led by the Sidney Kimmel Comprehensive Cancer Center at Johns Hopkins (NCT01088789).

The combined use of GVAX and iplimumab (anti-CTLA-4) in a cohort of patients with metastatic PDAC previously receiving FOLFIRINOX, was evaluated in another randomised phase II study (NCT01896869) which reported findings only recently [173]. In this cohort which was composed of two arms: iplimumab plus GVAX (arm A) versus continuation chemotherapy (FOLFIRINOX) (arm B), inferior survival of 9.38 months (95% CI, 5.0–12.2) versus 14.7 months (95% CI, 11.6–20.0) were reported. Furthermore, differentiation and augmentation of anti-tumour T-cell and macrophage (M1) responses were observed in arm A of the trial. Taken together with the aforementioned findings reported by Lutz et al., the combination of GVAX with iplimumab was superior to iplimumab monotherapy, however, inferior to FOLFIRINOX as maintenance therapy [173]. The combination of GVAX with single (nivolumab) or several other checkpoint inhibitors (nivolumab, iplimumab, urelumab and pembrolizumab), cyclophosphamide with or without radiotherapy in surgically resected cases, is currently evaluated in four phase II trials (NCT03161379, NCT03190265, NCT02451982, NCT02648282). All four are expected to complete enrolment and report findings between 2021 and 2023.

The C-C motif chemokine ligand-2 (CCL2) also enables pancreatic cancer immune evasion. The CC receptor 2 (CCR2)/CCL2 axis facilitates inflammatory cell mobilisation and suppressant macrophage (CCR2^+^) recruitment, further promoting a non-immunogenic TME [174]. The CCR5/CCL5 axis, apart from having a similar immunomodulatory role to CCR2/CCL2, has also been implicated in cancer progression and metastasis as well as a marker of poor prognosis in several cancers. Phase I/II trials evaluating clinical outcomes following nivolumab and a dual CCR2/CCR5 antagonist (BMS-813160) together with GVAX with (NCT03184870) and without (NCT03767582) chemotherapy, in locally advanced and metastatic PDAC, respectively, are currently recruiting.

#### 7.4.3. CRS-207

CRS-207 is a bacterial based vaccine of attenuated *Listeria monocytogenes* which was modified to express mesothelin (MSLN)—A tumour associated antigen (cell-surface glycoprotein) expressed in PDAC. MSLN facilitates cancer cell attachment on mesothelial surfaces, MMP upregulation and autocrine signalling—playing multiple roles in PDAC metastasis [175]. Early favourable results reported in a phase II study (NCT01417000) of 90 patients with metastatic PDAC [176], who previously had a combination of cyclophosphamide and GVAX, were randomly assigned to further receive Cyclo/GVAX or four doses of CRS-207, were not translatable in a larger phase IIb cohort study. The authors reported an OS of 9.7 months with CRS-207 versus 4.6 months in the Cyclophosphamide/GVAX arm [176]. The ECLIPSE study (NCT02004262) however, evaluated disease response in 303 patients with similar disease stage, to single agent chemotherapy, CRS-207 alone or CRS-207 plus Cyclo/GVAX. This larger, three-armed randomised phase IIb clinical trial failed to reach its primary efficacy point with OS of 3.7 (2.9–5.3), 5.4 (4.2–6.4), and 4.6 (4.2–5.7) months in these arms, respectively. No significant difference between chemotherapy and the combined treatments (HR = 1.17; 95% CI, 0.84–1.64) were observed [177].

#### 7.4.4. GI-4000

GI-4000 (GlobeImmune Inc., Louisville, CO, USA) is composed of recombinant yeasts which are engineered to express mutant KRAS epitopes in order to stimulate DCs, that have previously demonstrated to elicit an immune response in vivo [178]. Mutated proteins are seen as antigens by CD8^+^ T-cells which illicit a specific cytotoxic response against cells expressing such targets [178]. A phase II study (NCT00300950) of 176 RAS mutant (mRAS) pancreatic cancer positive subjects, randomised (1:1) patients to receive gemcitabine or gemcitabine in combination with GI-4000. A priming injection (or placebo) was given prior to gemcitabine cycles until the primary endpoints were reached. An anti mRAS (G12 codon mutation)-specific immune response (INFγ) was measured in patients with microscopically positive tumour resection margins (R1), as opposed to those with an R0 (with negative margins). When compared to those receiving the placebo (8.3%; 1/12), 46% of patients with R1 resection (7/15) demonstrated a significant response (*p* = 0.043) to gemcitabine plus GI-4000. Interestingly, longer median survival rates were observed in RAS mutated patients randomised to receive GI-4000 (by 773 days) than those which did not bear the mutation. With respect to RAS G12 mutated subjects receiving GI-4000 vs. placebo, those that were treated with GI-4000 showed a median survival which was longer by 568 days. In a subset of tumours with a specific proteomic signature (BDX-001) in this study, GI-4000 was shown to be of particular benefit. There are currently no actively recruiting trials in which GI-4000 is being further evaluated; however, a phase I (NCT03552718) trial will assess a novel yeast based vaccine (YE-NEO-001), in a subset of patients with pancreatic, colon and lung cancers who are in surveillance following a potentially curative treatment.

### 7.5. Adoptive Cell Therapies

Tumour infiltrating lymphocytes (TIL) are often found in surgically resected specimens of PDAC. As part of an anti-tumour immune response, infiltration by CD8^+^ and CD4^+^ T-cells results in recognition of tumour associated antigens presented on cancer cell surface using MHC-1 molecules, and a subsequent antitumour cytotoxic response. Density of T-cell infiltration has been directly correlated with improved survival in pancreatic cancer [179]. In TIL therapy, tumour reactive T-cells are isolated, expanded ex-vivo and genetically programmed to target specific antigens. T-cells can be “reprogrammed” by means of modifications applied to either T-cells with antigen specific receptors (TCRs) or a genetic modification that induces expression of chimeric antigen receptors (CAR) of interest. While TCRs are natively expressed by T-cells and are MHC presentation dependent, CARs are artificially induced and can both recognise specific antigens as well as further activate T-cells—without the need for antigen presentation using MHC molecules [180]. MHC-1 are able to present intracellularly degraded peptides, while CARs are only designed to recognise specific cell surface antigens.

MHC-1 expression, however, is often lost in cancer cells which challenges immune mediated tumour rejection using the TCR approach [181]. Despite numerous reports of successful use of TILs in treatment of mostly haematologic malignancies, their use in solid tumours however is underdeveloped [180]. Encouraging clinical response observed (complete remission) in ~22% of patients with metastatic melanoma that were treated using TIL infusions, have motivated their application in other cancers [182]. The expansion of TILs ex-vivo has been successful in several solid tumours (e.g., pancreatic, melanoma, ovarian, breast, and lung) and showed significant anti-tumour reactivity in preclinical and early clinical studies of TILs [182]. Specifically, in pancreatic cancer, isolation yield and anti-PDAC reactivity of TILs (CD4^+^, CD8^+^) can be improved by adjuvant use of immune checkpoint blockade (PD-1). TILs have been modified to recognise several neoantigens (including CEA, MUC-1, MSLN, prostate stem cell antigen (PSCA), claudin 18.2 and CD47, among others) in PDAC [183]. Despite promising results in pre-clinical study reports, the translation of such findings in clinical settings is still challenging. A phase I (NCT01212887) trial that evaluated anti-CEA CAR-T (CEACAM5^+^) treatment for CEA positive solid tumours, including PDAC, was halted due to observed acute respiratory toxicity, resulting from T-cell response directed against CEA co-expressing pulmonary epithelium.

In another study described by Beatty et al., the use of mRNA-based CAR-T generated T-cells directed against MSLN, in six patients with metastatic PDAC, showed stable disease in 30% of patients. In another case, the compassionate use of this approach in one patient with advanced PDAC, resulted in treatment tolerance as well as an objective evidence of antitumour humoral immune response. Multiple phase I/II studies of CAR-T treatment in pancreatic cancer are currently active (NCT04348643, NCT04581473, NCT04348643). A phase II trial (NCT04037241) is currently evaluating the benefit of combining anti-CEA CAR-T with different regimens of gemcitabine, nab-paclitaxel and capecitabine in 167 patients with liver metastatic lesions of CEA positive PDAC.

## 8. Targeting the Tumour Microenvironment

A hallmark of pancreatic cancer is the presence of a dense desmoplastic stroma, also known as the tumour microenvironment (TME), which can comprise up to 90% of the tumour volume. This stroma is composed of heterogeneous populations of cells, including cancer cells but also stromal components (e.g., fibroblasts, stellate cells, endothelial, neuronal, and immune cells) which are tightly embedded in a fibrotic extracellular matrix (ECM). This abundant ECM mediates elevations in interstitial fluid pressure and solid stress, thereby resulting in blood vessel constriction and vasculature collapse, reducing micro-vessel density and increasing intra-tumour hypoxia [10]. Several studies have confirmed the key role of the TME in PDAC disease progression, metastasis niche formation and therapeutic resistance by favouring immune evasion or acting as a mechanical barrier for drug delivery [184]. In the last years, it has also been shown the presence of cancer-associated fibroblast (CAF) phenotypic and functional heterogeneity (e.g., tumour-restraining and tumour-promoting CAFs), making the development of therapies targeting the crosstalk between cancer cells and stromal components challenging [185]. Thus, stromal-targeting therapy in PDAC is an emerging research field aiming at achieving the optimal balance between stromal depletion and TME reprogramming. In this section, we have summarised the main clinical trials currently evaluating this strategy.

A phase Ib/II open-label, single arm clinical trial (NCT04203641) is evaluating the TME modifier L-DOS47 in combination with doxorubicin, in advanced pancreatic cancer patients previously treated. L-DOS47 is a urease conjugated to an anti-CEACAM6 (carcinoembryonic antigen-related cell adhesion molecule 6) monoclonal antibody, which exploits the acidic TME to locally increase pH and produce ammonia toxicity. CEACAM6 is overexpressed in PDAC cases and is linked to poor prognosis. A phase I trial has already shown promise using L-DOS47 to treat NSCLC (NCT02309892) [186] and the phase I/II study has just been completed (NCT02340208).

The PDAC TME is characterised by high levels of hyaluronic acid (HA) that elevates interstitial pressure and impairs perfusion. A phase II study (HALO-109-202) evaluated the HA modulator PEGPH20 (pegylated recombinant human hyaluronidase), in combination with gemcitabine and nab-paclitaxel, compared with chemotherapy alone, in patients with metastatic untreated PDAC (NCT01839487) [187]. Promising results led to a subsequent phase III study (NCT02715804) testing the same combination. However, in this study, PEGPH20+gemcitabine+nab-paclitaxel did not significantly improve OS. PEGPH20 was also tested in a phase Ib/II trial in combination with mFOLFIRINOX versus mFOLFIRINOX alone, in patients with metastatic PDAC (NCT01959139). The addition of PEGPH20 to mFOLFIRINOX caused increased toxicity and treatment duration needed to be decreased compared to mFOLFIRINOX alone [188].

Different phase II trials have also evaluated inhibiting the Hedgehog signalling pathway as it regulates stroma deposition. However, compared to the promising results observed in preclinical studies, only a few targeted drugs improved patient survival, and their use was often accompanied by drug-related toxicity. For example, IPI-926 (NCT01130142) failed to show significant therapeutic benefits in PDAC patients. Similarly, vismodegib (Erivedge^®^, Genentech, South San Francisco, CA, USA), the first FDA-approved small molecule inhibiting the hedgehog pathway, was used in combination with gemcitabine in a randomised phase I/II trial (NCT01064622). The combination did not improve OS or PFS in patients with recurrent or metastatic pancreatic cancer compared to gemcitabine alone [189].

In addition to the above-mentioned drugs, other drugs targeting the stroma in phase II-III clinical trials include connective tissue growth factor (CTGF) antagonists, such as pamrevlumab (FG-3019; an anti-fibrotic agent). The randomised, double-blind phase III trial NCT03941093, is evaluating a neoadjuvant treatment with pamrevlumab or placebo in combination with gemcitabine plus nab-paclitaxel, for the treatment of locally advanced, non-resectable pancreatic cancer patients. CTGF antagonists have shown better results than anti-fibrotic agents (e.g., pirfenidone, still in preclinical stage) or metalloproteinase inhibitors (e.g., marimastat). A promising approach, still in phase I/II trials, is galectin targeted therapy. Galectins are members of the lectin family, and play a fundamental role in proliferation, metastasis, immunomodulation and angiogenesis. One galectin inhibitor specifically targeting galectin-9 is the monoclonal antibody LYT200. LYT200 alone or in combination with anti-PD1 or chemotherapy (gemcitabine+nab-paclitaxel), will be evaluated in a recently opened phase I/II open-label, multi-centre study (NCT04666688) in patients with relapsed/refractory metastatic solid tumours, including pancreatic cancer.

## 9. Emerging Therapies

A large proportion of the therapeutics currently being trialled for the treatment of pancreatic cancer are relatively well-known drugs, targeting key features of tumours such as angiogenesis or the TME, and key signalling pathways, as previously detailed. Moreover, the use of these therapeutics for the treatment of other cancers offers a reasonably understanding of their mechanism of action. However, due to the limited efficacy achieved with the currently available therapeutics, novel approaches targeting pancreatic cancer hallmarks and aiming at improving patient outcomes are incredibly relevant, providing innovative areas for further studies and novel combination therapeutic strategies that could potentially bring promising patient responses to treatment. Here we have summarised the most promising candidates in early stage clinical trials for pancreatic cancer.

### 9.1. OMT 110/111

Cancer cells use extensive amounts of glucose due to their abnormally high proliferation rates and elevated energy requirements. Under normal conditions, glucose would be metabolised via glycolysis and subsequently oxidised in the tricarboxylic acid (TCA) cycle. However, the elevated levels of glycolysis observed in cancer cells result in lactic acid production (Warburg effect). As this effect is commonly observed in solid tumours, regardless of their mutation status, different approaches have been explored to inhibit overexpressed glycolytic enzymes (such as lactate dehydrogenase A and pyruvate kinase M2), including the use of novel therapeutics such as OMT-110/111 [190].

OMT-110/111 promotes normal metabolism in cancer cells by activating the pyruvate dehydrogenase kinase (PDK) and subsequently the TCA cycle. This leads to a reduction in HIF-1 α, VEGF, and GLUT-1 (glucose transport channel) levels, decreasing pathological angiogenesis and glucose consumption, thus inhibiting tumour growth. Due to the novelty of OMT-110/111, not much is known about its efficacy in cancer treatment. However, one phase I study has used OMT-110 in patients with refractory colorectal cancer resistant to standard therapies [191]. Results showed no discontinued treatment, toxicity-mediated dose reductions, or serious adverse events following treatment with OMT-110. In addition, it was found that 100% of patients (nine out of nine) had stable metabolic disease or higher at eight weeks, with four patients demonstrating strong tendencies for reductions in glucose transporting. Therefore, OMT-110 appears to be safe and further investigations are undergoing. One phase II clinical trial is using OMT-110/111 for the treatment of terminal stage solid tumours resistant to standard therapy (metastatic triple negative breast cancer, advanced/metastatic pancreatic cancer, and metastatic NSCLC) (NCT04520386).

### 9.2. CG200745 (Ivaltinostat)

Histone epigenetic modifications are essential in mediating normal gene transcription and occur via histone acetyltransferases, that regulate histone acetylation, or histone deacetylases (HDACs), that remove histone acetyl groups. These processes are often dysregulated in cancer cells, with hyper-acetylation of oncogenes and hypo-acetylation of tumour suppressor genes observed [192]. Inhibition of HDACs has therefore been explored as a cancer treatment, with two inhibitors—vorinostat and romidepsin—previously receiving FDA approval for the treatment of T-cell lymphoma. Another novel HDAC inhibitor, CG200745 (ivaltinostat), has also demonstrated anti-tumour effects as a monotherapy or in combination with chemotherapy in various cancers including renal, colon, and pancreatic cancer [192]. CG200745 seems to enhance histone acetylation of the tumour suppressor p53, resulting in p53 protein accumulation ultimately inducing apoptosis of tumour cells [193]. A preclinical study by Lee et al. showed growth inhibition and anti-neoplastic effects in gemcitabine-resistant pancreatic cancer cells, following treatment with CG200745 plus gemcitabine and erlotinib (an EGFR inhibitor) in vitro and in vivo [193]. One phase I/II study explored the clinical effects of this combination (CG200745 plus gemcitabine and erlotinib) in 34 patients with unresectable, locally advanced/metastatic pancreatic cancer (NCT02737228) [194]. Part I consisted of 10 patients and set out to outline the maximum tolerated dose of CG200745, with results determining this to be 250 mg/m^2^. Phase II treated 24 patients with this dose of CG200745, plus 1000 mg/m^2^ of gemcitabine, and 100 mg/day of erlotinib. The OSR was found to be 25%, with a disease control rate of 93.8%. Eleven patients achieved stable disease and four partial responses were also observed. Despite several adverse events such as vomiting, neutrophil/platelet count reductions, and pyrexia being reported, this combination seems to be a promising option for the treatment of pancreatic cancer. Phase III trials are expected to be conducted [195].

### 9.3. Selinexor (KPT-330, XPOVIO)

The nuclear export protein, exportin 1, mediates transport of proteins from the nucleus to the cytoplasm and has been identified as a promising therapeutic target in cancer treatment. Selinexor (KPT-330, XPOVIO, Karyopharm Therapeutics, Newton, MA, USA) binds to exportin 1 to prevent the transport of tumour suppressor proteins from the nucleus to the cytoplasm. This results in retention and activation of tumour suppressor proteins in the nucleus, initiating cell cycle arrest and apoptosis in malignant cells [196]. Selinexor has shown promising results in a phase Ib/II clinical trial for the treatment of 18 relapsed/refractory multiple myeloma patients that had received prior treatment (including bortezomib, lenalidomide, pomalidomide, and daratumumab)(NCT02343042) [197]. Another phase IIb study treated 127 patients with relapsed/refractory diffuse large B-cell lymphoma with selinexor (NCT02227251). An ORR of 28% was observed, with 12% patients achieving a complete response and 17% a partial response [198]. Based on these promising results, the combination of selinexor, gemcitabine, and nab-paclitaxel is been tested in one phase Ib/II partially randomised clinical study (NCT02178436) for the treatment of nine metastatic PDAC patients. The phase I is now completed with two patients showing partial response, two stable disease, and one durable objective response to treatment (OS 22 months) [199]. Phase II is currently ongoing.

### 9.4. MVT-5873 (HuMab-5B1)

Carbohydrate antigen 19-9 (CA19-9) is a tumour marker overexpressed on the surface of pancreatic cancer cells. The Sialyl Lewis A antigen is a proteoglycan epitope on CA19-9, acting as a ligand for E selectin, enabling tumour cell invasion and metastasis [200]. Due to the limited expression of CA19-9 on normal cells, its targeting seems a promising treatment option. MVT-5873 (HuMab-5B1) is a human-derived IgG1 antibody specifically targeting the Sialyl Lewis A antigen on CA19-9 [201]. A phase I clinical trial is exploring the use of MVT-5873 for advanced pancreatic cancer, and other CA19-9-positive malignancies, either as a monotherapy or in combination with standard of care chemotherapy (gemcitabine/nab-paclitaxel) (NCT02672917) [202]. MVT-5873 is also being explored as a perioperative treatment for CA19-9-positive malignancies (including pancreatic cancer) in a phase II trial (NCT03801915).

### 9.5. Anetumab Ravtansine (BAY 94-9343)

Mesothelin is a cell surface antigen overexpressed in most pancreatic adenocarcinomas [203]. The biological function of this antigen is not entirely understood, although it is thought to contribute to pancreatic tumorigenesis by mediating constitutive activation of NF-κB/Akt, thus conferring resistance to TNF-α. It has also been associated with PI3K pathway activation and therefore its overexpression in pancreatic cancer may mediate cellular survival, proliferation, angiogenesis and chemotherapeutic resistance. As the expression of mesothelin on normal cells is limited, targeting of this antigen may prove promising as a treatment in pancreatic cancer.

The human anti-mesothelin antibody-drug conjugate, anetumab ravtansine (BAY 94-9343), has been developed as an anti-cancer therapeutic. Following anetumab-mediated targeting of mesothelin-expressing cells, the conjugated drug (DM4) binds to tubulin to disrupt microtubule assembly and prevent cell growth [203]. One ongoing phase Ib multi-indication study is using anetumab ravtansine in combination with gemcitabine in patients with mesothelin-expressing advanced pancreatic cancer (NCT03102320) [204]. Another phase II trial evaluating anetumab ravtansine in pre-treated (one or two prior chemotherapy regimens) mesothelin-expressing advanced pancreatic cancer has been completed (NCT03023722). However, results are not published yet. Moreover, the ongoing phase I/II study NCT03816358 is assessing anetumab ravtansine in combination with gemcitabine, ipilimumab (an anti- CTLA-4 antibody) and nivolumab (an anti-PD-1 antibody) in patients with mesothelin-expressing advanced/metastatic pancreatic cancer.

### 9.6. BT8009-100

Nectin-4 is a cell adhesion molecule that is overexpressed in pancreatic cancer and has been associated with poor prognosis [205]. One study by Nishiwada et al. demonstrated correlations between Nectin-4 and Ki67 (a cell proliferation marker) expression, and nectin-4 and VEGF expression in tissues from 123 PDAC patients [206]. This suggests that nectin-4 may play a role in pancreatic cancer cell proliferation and angiogenesis, respectively. Targeting of nectin-4 is therefore being explored with the bicycle toxin conjugate, BT8009-100, in a phase I/II clinical trial (NCT04561362). This study includes BT8009-100 as a monotherapy or in combination with nivolumab (an anti-PD-1 antibody) in patients with nectin-4-expressing advanced solid tumours, including pancreatic cancer.

## 10. Conclusions

Pancreatic cancer outcomes have minimally improved over the last years. In particular, PDAC is characterised by a high level of intra- and inter-tumour heterogeneity, making each tumour unique and representing a challenge for conventional therapies. We are in the era of personalised medicine, which has brought survival improvements in other cancers such as breast cancer, lung cancer, or melanoma. However, molecular profiling for pancreatic cancer patients has only been recently implemented. This may allow the identification of individuals with actionable alterations and offer them matched therapy. The information contained in this review summarises the current landscape of phase II and III clinical trials exploring the use of targeted agents in pancreatic cancer. Promising results and several emerging strategies pave the way to accelerate the design of novel multimodal targeted strategies which may allow more tailored therapies.

## Figures and Tables

**Table 1:** Ongoing clinical trials targeting the HER2 receptor.

Drug	Phase	Primary Outcome	Status	ID
Trastuzumab	II	ORR (12 months)	Recruiting	NCT04482309
Trastuzumab	I/II	RP2D, ORR (12 months)	Not yet recruiting	NCT04464967
Zenocutuzumab	I/II	AE, SAE, ORR (36 months), DOR (36 months), correlation of anti-tumour activity and biomarkers	Recruiting	NCT02912949
Afatinib	I/II	AE, PK, ORR (20 months)	Recruiting	NCT03785249

ORR, objective response rate; RP2D, recommended phase II dose; AE, adverse events; SAE, serious adverse events; DOR, duration of response; PK, pharmacokinetics.

**Table 2:** Other clinical trials targeting the somatostatin receptor in pancreatic cancer.

Drug	Phase	Primary Outcome	Status	ID
^177^Lu-Dotatate	II	PFS (72 months)	Not recruiting	NCT02736448
^177^Lu-Dotatate	I/II	ORR (24 months), toxicity rate	Not recruiting	NCT02736500
^177^Lu-Dotatate	II	DCR (7 years), acute toxicity	Not recruiting	NCT02489604
Octreotate	II	PFS (12/24 months)	N/S	NCT02358356
^177^Lu-DOTA^0^-Tyr^3^-Octreotate	II	PFS (12 months)	Recruiting	NCT02230176

PFS, progression free survival; ORR, objective response rate; DCR, disease control rate; N/S, not specified.

**Table 3:** Other receptors currently studied in clinical trials for the treatment of pancreatic cancer.

Drug	Target	Combination	Phase	Primary Outcome	Status	ID
CAB-AXL-ADC	AXL	-	I/II	AE, DLT, MTD, ORR	Recruiting	NCT03425279
Relacorilant	Glucocorticoid receptor	Nab-paclitaxel	III	ORR (24 months)	Recruiting	NCT04329949
CX-2029	CD71	-	I/II	DLT	Recruiting	NCT03543813
GEN1029	Death receptor 5	-	I/II	DLT, AE	Recruiting	NCT03576131
CORT125134	Glucocorticoid receptor	Nab-paclitaxel	I/II	MTD	Completed	NCT02762981
Ibrutinib	Bruton’s tyrosine kinase	Gemcitabine, Nab-paclitaxel	I/II	DLT, MTD	Not recruiting	NCT02562898
Tisotumab vedotin	Tissue factor	-	II	ORR (1 month)	Recruiting	NCT03485209
Entrectinib	Tropomysin receptor kinase	-	II	ORR (24 months)	Recruiting	NCT02568267

AE, adverse events; DLT, dose limiting toxicity; MTD, maximum tolerated dose; ORR, objective response rate.

**Table 4:** Anti-angiogenic drugs in ongoing clinical trials for the treatment of pancreatic cancer and their respective targets.

Drug	Combination	Phase	Primary Outcome	Status	ID
Apatinib	Camrelizumab	II	ORR (2 years)	Recruiting	NCT04415385
Apatinib	Irinotecan plus S-1	II	PFS (1 year)	Recruiting	NCT04101929
Ramucirumab	mFOLFIRINOX	II	PFS (9 months)	Not recruiting	NCT02581215
AZD4547	-	II	ORR (3 years)	Recruiting	NCT02465060
Erdafitinib	-	II	ORR (3 years)	Recruiting	NCT02465060
Cetuximab	SNK01	I/II	RP2D, ORR (12 months)	Not yet recruiting	NCT04464967
Cetuximab	MRTX849	I/II	Safety, PK, clinical activity	Recruiting	NCT03785249
Bevacizumab	NANT	I/II	AE, SAE, ORR (1 year)	Not recruiting	NCT03387098
Bevacizumab	Durvalumab, CV301, capecitabine	I/II	RP2D, PFS (8.5 months), PFS (4 months)	Not recruiting	NCT03376659
Bevacizumab	NANT	I/II	AE, SAE, ORR (1 year)	Not recruiting	NCT03329248
Bevacizumab	Gemcitabine, nab-paclitaxel, atezolizumab	I/II	ORR (3–5 years), AE	Recruiting	NCT03193190
Bevacizumab	NANT	I/II	AE, SAE, ORR (1 year)	Not recruiting	NCT03136406
Bevacizumab	Capecitabine, temozolomide	II	RR (18 months)	Not recruiting	NCT01525082
Bevacizumab	Octreotide acetate, everolimus	II	PFS (3 years)	Not recruiting	NCT01229943
TS-1	Gemcitabine	II	DFS (24 months)	Recruiting	NCT02754180
Nintedanib	-	I/II	MTD	Recruiting	NCT02902484
Pazopanib	-	II	PFS (5 years)	Not recruiting	NCT01841736
Pazopanib	Temozolomide	I/II	MTD, RP2D, ORR (2 months)	Not recruiting	NCT01465659
Regorafenib	-	II	PFS (2 months)	Not recruiting	NCT02307500
Regorafenib	-	II	PFS (6 months)	Not recruiting	NCT02259725
Surufatinib	-	III	PFS (7 months)	Not recruiting	NCT02589821
Pamrevlumab	Gemcitabine, nab-paclitaxel	III	OS (18 months), R0/R1 resections	Recruiting	NCT03941093
Pamrevlumab	Gemcitabine, nab-paclitaxel	I/II	Safety, R0/R1 resections, TRR (24 weeks), OS, PFS (52 weeks)	Not recruiting	NCT02210559
ENB003	Pembrolizumab	I/II	AE, ORR (2 years)	Not yet recruiting	NCT04205227

ORR, objective response rate; PFS, progression free survival; RP2D, recommended phase II dose; PK, pharmacokinetics; AE, adverse events; SAE, serious adverse events; RR, radiographic response; DFS, disease free survival; MTD, maximum tolerated dose.

**Table 5:** Current phase I/II, II and III clinical trials using PARP inhibitors for the treatment of pancreatic cancer.

Drug	Combination	Phase	Primary Outcome	Status	ID
Niraparib	-	II	ORR (8 weeks)	Recruiting	NCT03553004
Niraparib	-	II	PFS (6 months)	Recruiting	NCT03601923
Niraparib	Dostarlimab	II	DCR (12 weeks)	Recruiting	NCT04493060
Niraparib	Dostarlimab, radiotherapy	II	DCR (2 years)	Recruiting	NCT04409002
Niraparib	Nivolumab or Ipilimumab	I/II	PFS (6 months)	Recruiting	NCT03404960
Rucaparib	Liposomal irinotecan, fluorouracil, leucovorin	I/II	DLT, ORR (3 years), BRR (32 weeks)	Recruiting	NCT03337087
Rucaparib	-	II	AE	Not recruiting	NCT03140670
Rucaparib	-	II	ORR (2 years)	Recruiting	NCT04171700
Veliparib	Gemcitabine hydrochloride + cisplatin	II	Dosing, ORR (5 years)	Not recruiting	NCT01585805
Veliparib	mFOLFIRI	II	OS (3 years)	Not recruiting	NCT02890355
Veliparib	mFOLFOX-6	I/II	DLT	Not recruiting	NCT01489865
Olaparib	-	II	ORR (24 weeks)	Not recruiting	NCT02677038
Olaparib	-	III	PFS (4 years)	Not recruiting	NCT02184195
Olaparib	Pembrolizumab	II	PFS (3 years)	Recruiting	NCT04548752
Olaparib	Cediranib	II	ORR (4 weeks)	Recruiting	NCT02498613
Olaparib	AZD6738 (Ceralasertib)	II	ORR (2.5 years)	Recruiting	NCT03682289
Fluzoparib	-	III	PFS (3 years)	Recruiting	NCT04300114
Fluzoparib	mFOLFIRINOX	I/II	DLT, MTD, ORR (24 months)	Recruiting	NCT04228601

ORR, objective response rate; PFS, progression free survival; DCR, disease control rate; DLT, dose limiting toxicity; BRR, best response rate; AE, adverse events; OS, overall survival; MTD, maximum tolerated dose.

**Table 6:** Therapeutics targeting JAK-STAT/Src pathway components and their activity in ongoing clinical trials for the treatment of pancreatic cancer.

Drug	Combination	Phase	Primary Outcome	Status	ID
Ruxolitinib	Capecitabine + regorafenib	II	AE, SAE	Enrolling by invitation	NCT02955940
Danvatirsen	Durvalumab	II	AE, SAE	Not recruiting	NCT02983578
Napabucasin	Gemcitabine + paclitaxel	III	OS (30 months)	Recruiting	NCT03721744
Dasatinib	mFOLFOX6	II	PFS (3 years)	Completed	NCT01652976
Dasatinib	-	II	ORR (3 years)	Recruiting	NCT02465060

AE, adverse events; SAE, serious adverse events; OS, overall survival; PFS, progression free survival; ORR, objective response rate.

**Table 7:** Therapeutics targeting MAPK/ERK pathway components in ongoing clinical trials for the treatment of pancreatic cancer.

Drug	Combination	Phase	Primary Outcome	Status	ID
AMG 510	-	I/II	ORR (24 months)	Recruiting	NCT03600883
Encorafenib	Binimetinib	II	ORR (24 weeks)	Not yet recruiting	NCT04390243
Dabrafenib	Trametinib	II	ORR (3 years)	Recruiting	NCT02465060
Cobimetinib	Atezolizumab	I/II	ORR (3–5 years), AE	Recruiting	NCT03193190
Cobimetinib	RMC-4630	I/II	AE, SAE, DLT	Recruiting	NCT03989115
Selumetinib	-	II	ORR (2 months)	Completed	NCT03040986
Binimetinib	-	II	ORR (3 years)	Recruiting	NCT02465060
Binimetinib	Avelumab and talazoparib	I/II	DLT, ORR (24 months)	Recruiting	NCT03637491
Trametinib	Dabrafenib	II	ORR (3 years)	Recruiting	NCT02465060
Trametinib	GSK2256098	II	ORR (24 weeks)	Not yet recruiting	NCT02428270
Ulixertinib	-	II	ORR (3 years)	Recruiting	NCT02465060

ORR, objective response rate; AE, adverse events; SAE, serious adverse events; DLT, dose limiting toxicity.

**Table 8:** Current phase II clinical trials using PI3K/AKT7mTOR inhibitors for the treatment of pancreatic cancer.

Drug	Combination	Phase	Primary Outcome	Status	ID
Seribantumab	-	II	ORR (12 months)	Recruiting	NCT04383210
ABTL0812	Gemcitabine plus nab-paclitaxel	I/II	AE	Not yet recruiting	NCT03417921
ABTL0812	FOLFIRINOX	II	RP2D (6 months), PFS (1 year), ORR (1 year)	Not yet recruiting	NCT04431258
Larotrectinib	-	II	ORR (3 years)	Recruiting	NCT02465060

ORR, objective response rate; AE, adverse events; RP2D, recommended phase II dose; PFS, progression free survival.

**Table 9:** Clinical trials testing immunotherapy agents for pancreatic cancer.

Drug	Combination	Phase	Primary Outcome	Status	ID
Iplimumab	Niraparib	Ib/II	PFS (6 months)	Recruiting	NCT03404960
Iplimumab	Cyclophosphamide, Nivolumab, GVAX, CRS207	II	ORR (4 years)	Recruiting	NCT03190265
Iplimumab	Nivolumab, radiotherapy	II	DCR (2 years)	Recruiting	NCT03104439
Iplimumab	Nivolumab, radiotherapy	II	CBR (6 months)	Recruiting	NCT02866383
Iplimumab	Nivolumab	II	ORR (10 years)	Recruiting	NCT02834013
Iplimumab	Nivolumab, radiotherapy	II	ORR (6 weeks)	Recruiting	NCT04361162
Iplimumab	Tocilizumab, Nivolumab, radiotherapy	II	ORR (12 months)	Recruiting	NCT04258150
Iplimumab	Gemcitabine, Nab-paclitaxel, Nivolumab, radiotherapy	II	AE, SAE	Recruiting	NCT04247165
Iplimumab	Gemcitabine, Anetumab, Nivolumab	II	MTD	Recruiting	NCT03816358
Tremelimumab	Gemcitabine, Durvalumab, MIS-MWA	II	PFS (12 months)	Recruiting	NCT04156087
Tremelimumab	Durvalumba, radiotherapy	II	AE	Not recruiting	NCT02311361
Plerixafor	Cemiplimab	II	ORR (4 years)	Recruiting	NCT04177810
BL-8040	Pembrolizumab	II	ORR (24 months)	Not recruiting	NCT02826486
BL-8040	Multiple chemotherapy, anti PD-1/PDL-1, MEK inhibitors, CD40 agonists, VEGF targeting agents, A2aR inhibitors, CXCR4 inhibitors and immune-cytokines	I/II	ORR (3–5 years), AE	Recruiting	NCT03193190
Selicrelumab	Multiple chemotherapy, anti PD-1/PDL-1, MEK inhibitors, CD40 agonists, VEGF targeting agents, A2aR inhibitors, CXCR4 inhibitors and immune-cytokines	I/II	ORR (3–5 years), AE	Recruiting	NCT03193190
Tocilizumab	Iplimumab, Nivolumab, radiotherapy	II	ORR (12 months)	Recruiting	NCT04258150
Tocilizumab	Gemcitabin, Nab-Paclitaxel	II	OS (6 months)	Recruiting	NCT02767557
Siltuximab	Spartalizumab	Ib/II	MTD	Recruiting	NCT04191421
N17 (efineptakin)	Pembrolizumab	Ib/IIa	MTD, RP2D, AE, DLT	Recruiting	NCT04332653
Oleclumab	Durvalumab, multiple chemotherapy	Ib/II	AE, ORR (2 years)	Recruiting	NCT03611556
Daratumumab	Nivolumab	I/II	AE, SAE	Not recruiting	NCT03098550
NIR178	PDR001	II	ORR (40 weeks)	Recruiting	NCT03207867

PFS, progression free survival; ORR, objective response rate; DCR, disease control rate; CBR, clinical benefit rate; AE, adverse events; SAE, serious adverse events; MTD, maximum tolerated dose; OS, overall survival; RP2D, recommended phase II dose; DLT, dose limiting toxicity.

**Table 10:** Clinical trials testing cancer vaccines and CAR-T based therapies for pancreatic cancer.

Approach	Combination	Phase	Primary Outcome	Status	ID
CV-301	Capecitabine, Durvalumab	I/II	RP2D, PFS (8.5 months)	Not recruiting	NCT03376659
GVAX	Nivolumab, Cyclophosphamide	II	CD8^+^ count in TME	Recruiting	NCT03161379
GVAX	Nivolumab, Iplimumab, Cyclophosphamide, CRS-207	II	ORR (4 years)	Recruiting	NCT03190265
GVAX	Nivolumab, Urelumab	II	IL17A expression	Recruiting	NCT02451982
GVAX	Pembrolizumab, Cyclophosphamide, Radiotherapy	II	DMFS (4 years)	Recruiting	NCT02648282
GVAX	BMS-813160 (CCR2/CCR5 antagonist), Nivolumab, multiple chemotherapy (Gemcitabine, Nab-Paclitaxel, 5-FU, Leucovorin, Irinotecan)	Ib/II	AE, SAE, ORR (2 years), DOR, PFS (24 weeks), decrease in Treg and TAMs	Recruiting	NCT03184870
GVAX	BMS-813160 (CCR2/CCR5 antagonist), Nivolumab, radiotherapy	I/II	DLT, CD8^+^ and CD37^+^ in TME	Recruiting	NCT03767582
CRS-207	Epacadostat, Pembrolizumab, GVAX, Cyclophosphamide	II	RP2D, OS (6 month)	Recruiting	NCT03006302
CRS-207	Nivolumab, Iplimumab, Cyclophosphamide, GVAX	II	ORR (4 years)	Recruiting	NCT03190265
Anti CEA CAR-T	-	I/II	AE	Recruiting	NCT04348643
Anti CEA CAR-T	Gemcitabine, Nab-Paclitaxel, NLIR + FU/FA	II	OS (12 months)	Not yet recruiting	NCT04037241
Anti Claudin18.2 CAR-T	-	Ib/II	AE, MTD, DLT, ORR (24 weeks)	Recruiting	NCT04581473

RP2D, recommended phase II dose; PFS, progression free survival; TME, tumour microenvironment; ORR, objective response rate; DMFS, distant metastasis free survival; AE, adverse events SAE, serious adverse events; DOR, duration of response; Treg, regulatory T cells; TAMs, tumour-associated macrophages; DLT, dose limiting toxicity; OS, overall survival; MTD, maximum tolerated dose.
